# Three new deep-sea species of Thyasiridae (Mollusca: Bivalvia) from the abyssal plain of the northwestern Pacific Ocean and hadal depths of the Kuril-Kamchatka Trench

**DOI:** 10.7717/peerj.10405

**Published:** 2020-11-25

**Authors:** Gennady M. Kamenev

**Affiliations:** A.V. Zhirmunsky National Scientific Center of Marine Biology, Far Eastern Branch, Russian Academy of Sciences, Vladivostok, Russian Federation

**Keywords:** Bivalvia, Taxonomy, Abyssal, Hadal, Kuril-Kamchatka Trench, Northwestern Pacific

## Abstract

The Thyasiridae is the most species-rich family of bivalves in the abyssal and hadal zones of the northwestern Pacific Ocean. In recent years, with at least 14 thyasirid species found in that region at depths exceeding 3,000 m. Some of them are the numerically dominant species in bottom communities. However, all members in that family have not yet been identified to the species level. Based on the material collected from 1953 to 2016 by five deep-sea expeditions, three new species of Thyasiridae (Mollusca: Bivalvia) are described from the abyssal and hadal zones of the northwestern Pacific. *“Axinulus” roseus* sp. nov. was found in the Kuril-Kamchatka Trench at 9,000–9,583 m depth. This species has a large rhomboidal shell with strong commarginal sculpture, a well defined, long and deep lunule and escutcheon without an auricle, a ctenidium consisting of a single demibranch, extensively lobed lateral pouches, and a large prodissoconch with specific sculpture. It is one of the dominant species in terms of abundance in macrobenthic communities in the deepest basin of the Kuril-Kamchatka Trench with a population density of up to 396 ind. m^−2^. The species has a shell length of up to 9.0 mm and it is the largest thyasirid with a single demibranch. *“Axinulus” oliveri* sp. nov. was found in a vast region of the northwestern Pacific on the abyssal plain adjacent to the Kuril-Kamchatka Trench, on the abyssal slope of the Kuril Islands, and in the Kuril-Kamchatka Trench at 4,648–6,168 m depth. This species is characterized by its ovate-rhomboidal shell, a well defined, deep and long escutcheon with a distinct auricle, a ctenidium with a single demibranch, and extensively lobed lateral pouches. It is widespread in the northwestern Pacific and forms populations with a density of up to 36 ind. m^−2^. Scanning electron microscopic observation of the gills of *“A.” roseus* sp. nov. and *“A.” oliveri* sp. nov. revealed that these species are not chemosymbiotic. *“Axinulus” roseus* sp. nov. and *“A.” oliveri* sp. nov. are provisionally assigned to the genus *Axinulus*, because they differ from the type species of the genus in a number of morphological and anatomical features. *Parathyasira fragilis* sp. nov. was found on the abyssal plain adjacent to the Kuril-Kamchatka Trench at 5,249–5,399 m depth. This species is distinguished by its very thin, fragile, dorsoventrally elongated, rhomboidal shell with very long anterodorsal margin and a long, wide, flat lunule. The taxonomic position of the new species is discussed.

## Introduction

The family Thyasiridae contains about 130 species (WoRMS Editorial Board, 2020) that are very widespread in the world’s oceans. Thyasirids were found in the northern and southern hemispheres, from the poles to the equator and from the intertidal to the maximum depths of the oceans (Filatova, 1961, 1968; Bernard, 1979; Scarlato, 1981; Belyaev, 1989; Payne & Allen, 1991; Coan, Scott & Bernard, 2000; Okutani, 2000; Oliver & Killeen, 2002; Allen, 2008, 2015; Zelaya, 2009, 2010; Coan & Valentich-Scott, 2012; Oliver et al., 2013, Oliver, 2015, Oliver & Rodrigues, 2017). This is the most diverse family among bivalves in the deep Atlantic. Seventy-nine species of thyasirids were recorded in the Atlantic at depths exceeding 500 m (24.5% of the total number of bivalve species) (Allen, 2008). Most of them were found in the bathyal zone at depths to 3,000 m. No less than 30 species of the Thyasiridae were recorded in the abyssal zone of the Atlantic Ocean at depths from 3,000 to 5,000 m. However, at depths greater than 5,000 m, only five species of thyasirids were recorded with only two of them being identified to the species level, *Adontorhina transversa* (Payne & Allen, 1991) and *Thyasira subovata* (Jeffreys, 1881) (Allen, 2008).

In the Pacific Ocean, abyssal plains and deep-sea trenches with depths greater than 5,000 m occupy vast areas (Belyaev, 1989). However, until recently no more than seven species of thyasirids were recorded in the Pacific Ocean at depths greater than 5,000 m (Belyaev, 1989; Okutani, Fujikura & Kojima, 1999; Okutani, 2000; Allen, 2015). All of them, except *Thyasira kaireiae* (Okutani, Fujikura & Kojima, 1999), were found exceptionally in the hadal zone (below 6,000 m) of oceanic trenches in the western Pacific. To date, only three of the seven species were described (*Axinulus hadalis* (Okutani, Fujikura & Kojima, 1999), *Axinulus philippinensis* Allen, 2015, and *T. kaireiae*). In the eastern Pacific, no species of the Thyasiridae were found at depth greater than 5,000 m (Coan, Scott & Bernard, 2000; Coan & Valentich-Scott, 2012).

Since 2010, German and Russian scientists have been actively exploring the deep-sea fauna of the northwestern Pacific Ocean. During the period from 2010 to 2016, four joint deep-sea expeditions have been performed that investigated the bottom fauna of deep-sea basins of the Sea of Japan (SoJoBio (Sea of Japan Biodiversity Studies) expedition, 2010) and the Sea of Okhotsk (SokhoBio (Sea of Okhotsk Biodiversity Studies) expedition, 2015), the abyssal plain of the Pacific Ocean adjacent to the Kuril-Kamchatka Trench (KuramBio (Kurile Kamchatka Biodiversity Studies) expedition, 2012), and the hadal zone of the Kuril-Kamchatka Trench (KuramBio II expedition, 2016) (Malyutina & Brandt, 2013; Brandt & Malyutina, 2015; Malyutina, Chernyshev & Brandt, 2018; Brandt et al., 2020). The studies of the northwestern Pacific deep-sea regions have revealed a rich fauna of bivalves represented by many families, with the Thyasiridae being predominant in species number (Kamenev, 2013, 2015, 2018, 2019). No less than 14 species of thyasirids were found at depths greater than 5,000 m on slopes of the Kuril Islands, at the Pacific abyssal plain, and in the Kuril-Kamchatka Trench, eight of them being recorded from depths below 6,000 m in the Kuril-Kamchatka Trench. Many thyasirids were found in samples in large numbers, being the dominant species of bottom communities on the floor of deep-sea basins of the Sea of Japan and the Sea of Okhotsk (below 3,000 m depth), on the oceanic slope of the Kuril Islands and at the abyssal plain adjacent to the Kuril-Kamchatka Trench (below 5,000 m depth), and on the floor of the Kuril-Kamchatka Trench (below 9,000 m depth). Earlier, various species of thyasirids were also recorded in large numbers from the floor of the Japan Trench, Kermadec Trench, and Java Trench (Belyaev, 1989). However, despite the high species richness and abundance, and correspondingly an important role in northwestern Pacific deep-sea ecosystems, none of the deep-sea Thyasiridae recently found by the joint Russian–German expeditions has so far been identified to the species level. This also relates to other thyasirids collected by previous Russian expeditions in the hadal zone of the various trenches of the Pacific Ocean (Belyaev, 1989). Preliminary examination showed that most are likely new to science. This work is an initial step in taxonomic investigations of numerous thyasirid species found in the abyssal and hadal zones of the northern Pacific. The present paper describes three new species of the Thyasiridae, which are relatively large-sized for deep-water fauna. Two of them are widely distributed at depths greater than 5,000 m on the abyssal plain adjacent to the Kuril-Kamchatka Trench. The third species was found in large numbers on the bottom of the Kuril-Kamchatka Trench at depths below 9,000 m, where it is one of the dominant species of macrobenthos.

## Materials and Methods

### Material studied

The material examined in this study was collected from 1953 to 1954 by the P.P. Shirshov Institute of Oceanology, Russian Academy of Sciences (IO RAS) expeditions adjacent to the Kuril-Kamchatka Trench and on the bottom of the Kuril-Kamchatka Trench (RV *Vityaz*, cruise no. 14, May 2–July 5, 1953; RV *Vityaz*, cruise no. 19, August 17–October 29, 1954), as well as by the German–Russian deep-sea expeditions KuramBio (RV *Sonne*, cruise no. 223, July 21–September 7, 2012) and KuramBio II (RV *Sonne*, cruise no. 250, August 16–September 29, 2016) at the Pacific abyssal plain adjacent to the Kuril-Kamchatka Trench and in the hadal zone of the Kuril-Kamchatka Trench and by the Russian-German deep-sea expedition SokhoBio in the abyssal zone of the Pacific slope of the Kuril Islands (RV *Akademik M.A. Lavrentyev*, cruise no. 71, July 6–August 6, 2015). New species of Thyasiridae were found in 34 samples at depths between 4,681 and 9,583 m. Sampling during the IO RAS expeditions was carried out using an Okean grab (sampling area of 0.25 m^2^) and Sigsbee trawl; the KuramBio, KuramBio II, and SokhoBio expeditions used a large box corer (sampling area of 0.25 m^2^), epibenthic sledge, and Agassiz trawl. All samples collected by the IO RAS were fixed in 4% buffered formaldehyde, later transferred to 70% ethanol, and stored in the IO RAS. Samples collected by the KuramBio, KuramBio II, and SokhoBio expeditions were fixed in pre-cooled 96% ethanol and 4% buffered formaldehyde and stored in the Museum of the Institute of Marine Biology, A.V. Zhirmunsky National Scientific Center of Marine Biology, Far Eastern Branch, Russian Academy of Sciences (MIMB), Vladivostok (Russia).

Further material was examined for the comparison purposes: *Axinulus careyi* Bernard, 1979 (holotype, LACM (Natural History Museum of Los Angeles County, Los Angeles, USA) 1990; paratype, SBNHM (Santa Barbara of the Natural History Museum, Santa Barbara) 55440; *Axinulus thackergeigeri* Valentich-Scott & Coan in Coan & Valentich-Scott, 2012 (holotype, SBNHM 149742; paratype, SBNHM 149743); *Axinulus obliqua* Okutani, 1968 (holotype, NSMT (National Museum of Nature and Science, Tsukuba, Japan) Mo 69719); *Clausina croulinensis* Jeffreys, 1847 (neotype, USNM (National Museum of Natural History) 62048, photos from USNM Web site); *Cryptodon (Axinulus) brevis* Verrill & Bush, 1898 (holotype, USNM 159873, photos from USNM Web site); *Cryptodon* (*Axinulus) simplex* Verrill & Bush, 1898 (holotype, USNM 159888, photos from USNM Web site); *Cryptodon equalis* Verrill & Bush, 1898 (holotype, USNM 74302, photos from USNM Web site); *Maorithyas hadalis* Okutani, Fujikura & Kojima, 1999 (paratype, NSMT Paratype C Mo 71432-c); *Parathyasira kaireiae* Okutani, Fujikura & Kojima, 1999 (holotype, NSMT Mo 71433); *Thyasira dearborni* Nicol, 1965 (holotype, USNM 653099, photos from USNM Web site); *Thyasira magellanica* Dall, 1901 (holotype, USNM 122745, photos from USNM Web site). Information and photos from USNM Web site provided with the permission of the National Museum of Natural History, Smithsonian Institution, 10th and Constitution Ave. N.W., Washington, DC 20560-0193 (http://www.nmnh.si.edu/).

### Shell measurements

**Figure 1:**
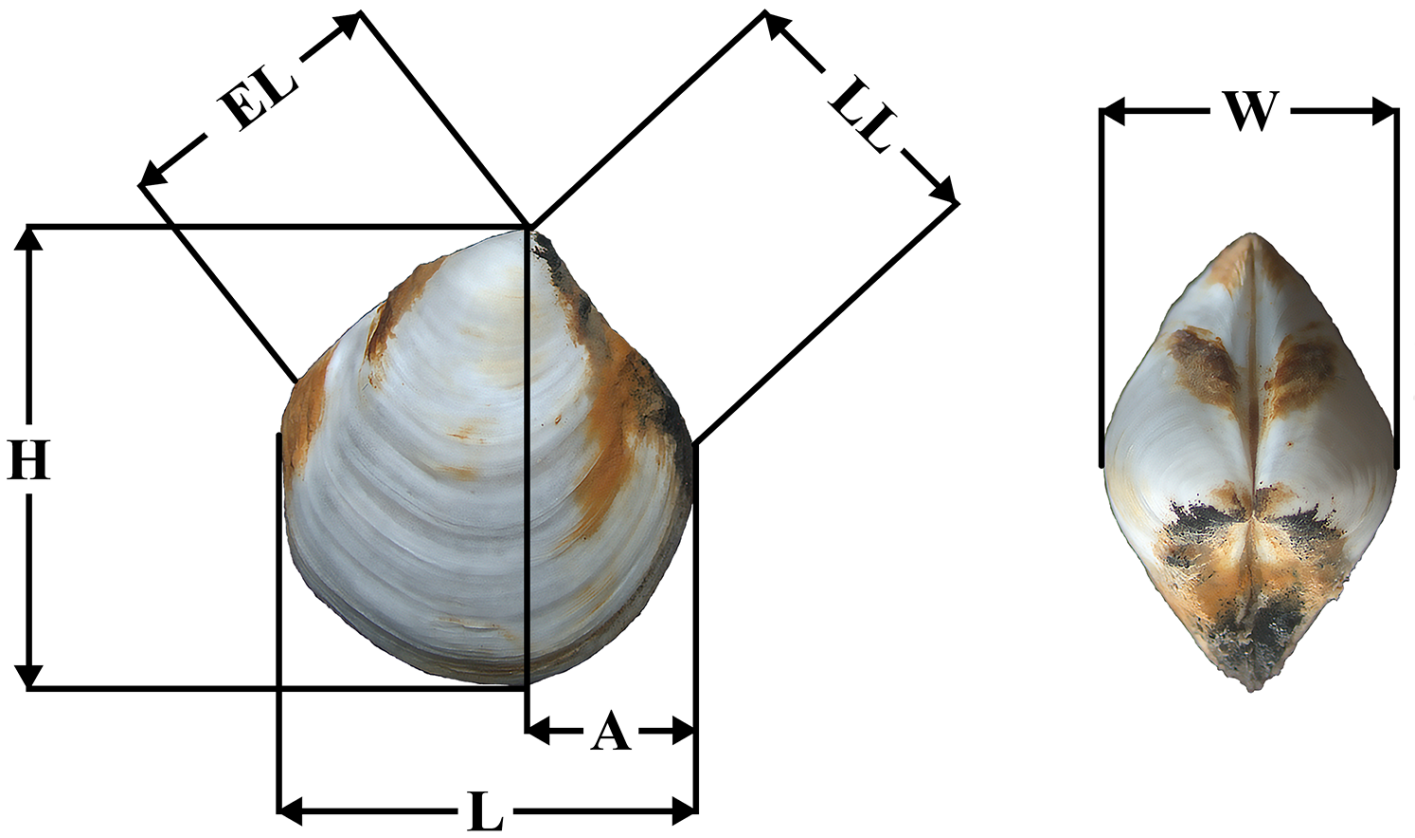
Placement of shell measurements. Abbreviations: *L*, shell length; *H*, height; *A*, anterior end length; LL, lunule length, EL, escutcheon length, *W*, shell width.

Figure 1 shows the shell morphology measurements. Shell length (L), height (H), anterior end length (A), lunule length (LL), escutcheon length (EL) and shell width (W) were measured for shells. The ratios of these parameters to shell length (H/L, A/L, LL/L, EL/L, W/L, respectively) were determined. Shell measurements were made using an ocular micrometer with an accuracy of 0.1 mm.

## Methods

For scanning electron microscopy, shells were cleaned of traces of soft tissues and periostracum in 50% diluted commercial bleach, washed in distilled water, and dried. They were then mounted to aluminium stubs using an adhesive tape and coated with chromium for examination with a SIGMA 300VP (Carl Zeiss, Cambridge, UK).

Gross anatomy was observed on preserved live-taken specimens. For anatomical studies, specimens of all species were dissected in 70% ethanol. For scanning electron microscopy, gills were transferred to distilled water and washed using a detergent to clean the gill surface. The cleaned gills were dehydrated through a graded series of ethanol, transferred to acetone, and critical-point dried. The dried gills were cut transversely and longitudinally using a thin razor blade, mounted to aluminium stubs using an adhesive tape and coated with chromium for examination with a SIGMA 300VP (Carl Zeiss, Cambridge, UK).

Microscopic observations of shells and bodies were performed in a Zeiss Discovery 8 stereomicroscope at the Far Eastern Center of Electron Microscopy of the A.V. Zhirmunsky National Scientific Center of Marine Biology, Far Eastern Branch, Russian Academy of Sciences (NSCMB FEB RAS). The terminology of the shell morphology and body anatomy of the family Thyasiridae follows Payne & Allen (1991), Oliver & Killeen (2002), and Oliver (2014).

### Nomenclatural acts

The electronic version of this article in Portable Document Format (PDF) will represent a published work according to the International Commission on Zoological Nomenclature (ICZN), and hence the new names contained in the electronic version are effectively published under that Code from the electronic edition alone. This published work and the nomenclatural acts it contains have been registered in ZooBank, the online registration system for the ICZN. The ZooBank LSIDs (Life Science Identifiers) can be resolved and the associated information viewed through any standard web browser by appending the LSID to the prefix http://zoobank.org/. The LSID for this publication is: urn:lsid:zoobank.org:pub:E693F0D2-D228-4E99-A7CD-143A5A7E2B9B. The online version of this work is archived and available from the following digital repositories: PeerJ, PubMed Central and CLOCKSS.

## Results

**Systematics**

**Class** Bivalvia

**Order** Lucinoida Gray, 1854

**Superfamily** Thyasiroidea Dall, 1900 (1895)

**Family** Thyasiridae Dall, 1900 (1895)

**Genus** *Axinulus* Verrill & Bush, 1898

Type species (by original designation): *Cryptodon (Axinulus) brevis* Verrill & Bush, 1898

**Diagnosis:** Shell small (<10 mm), fragile, equivalve, equilateral or subequilateral, ovate to ovate-rhomboidal, slightly higher than long; margins entire, without any posterior sinus, but sometimes with weak angulation of posterior margin; posterior sulcus absent or weak. Lunule and submarginal sulcus variably expressed, indistinct to deep; escutcheon and auricle sometimes present. Hinge plate edentulous, sometimes with a small swelling, ligament sunken. Ctenidium of a single demibranch, foot vermiform, lateral body pouches without or with distinct lobes.

**Remarks:** According to WoRMS (WoRMS Editorial Board, 2020), the genus *Axinulus* currently contains 12 species. Nevertheless, Zelaya (2010), Oliver (2015), and Allen (2015), who recently examined species of the genus and described new species think that only 5 species unequivocally belong to the genus *Axinulus*: *Axinulus alleni* (Carrozza, 1981), from the Mediterranean Sea, Angola, and Cape Basins; *Axinulus brevis* (Verrill & Bush, 1898) and *Axinulus croulinensis* (Jeffreys, 1847), from the northern Atlantic Ocean (American, European, and African coasts) and the southern Atlantic Ocean (Angola, Cape, and Argentine Basins, with the southernmost record at ~37°S) (Payne & Allen, 1991; Janssen & Krylova, 2014) (*A. croulinensis* apparently also occurs in the abyssal zone of the Indian Ocean (Arabian Sea) (Oliver, 2015)); *Axinulus antarcticus* Zelaya, 2010, from the Antarctic waters (from the Ross Sea to South Orkneys, 68–850 m) (Zelaya, 2010); and *A. philippinensis*, from the Philippine Trench (9,605–9,807 m) (Allen, 2015). Furthermore, Huber (2015) assigned *Axinulus subequatorius* (Payne & Allen, 1991) to the genus *Axinulus*; originally, the species was described as *Thyasira (Parathyasira) subequatoria* Payne & Allen, 1991 from the south Atlantic Ocean (Angola and Argentine Basins) (Payne & Allen, 1991). The shell morphology and anatomy of all the above 6 species were investigated and they all share a number of common features: a small shell size (length less than 5 mm); shell height greater than length; indistinct lunule, auricle and submarginal sulcus; nonraised adductor scars; absence of lateral teeth; presence of a single demibranch; foot without heel (Payne & Allen, 1991; Oliver & Levin, 2006; Zelaya, 2010; Allen, 2015; Oliver, 2015). Therefore, a comparative analysis is made between the new species proposed in the present study and only the above species.

As regards the remaining six species of the genus *Axinulus* that are registered in the WoRMS, their shell size, proportions, and morphology differ markedly from those of the type species of the genus *Axinulus*, while the anatomy of the species was not investigated. Oliver (2015) thinks that these species do not conform and need reassignment or placement in as yet undescribed genera. According to Oliver’s opinion, *A. careyi* has the appearance of *Mendicula* and *A. hadalis* and *A. thackergeigeri* requires novel placement. After examining the type material of the 6 species, I agree with the point of view that their assignment to the genus *Axinulus* is doubtful. The anatomy of these species remains unstudied and the type material is represented merely by shells. The size, shape, proportions, and morphology of the shell in most of the species differ markedly from those of the type of *Axinulus*. In all the species, the shell height is smaller than the shell length.

**Figure 2:**
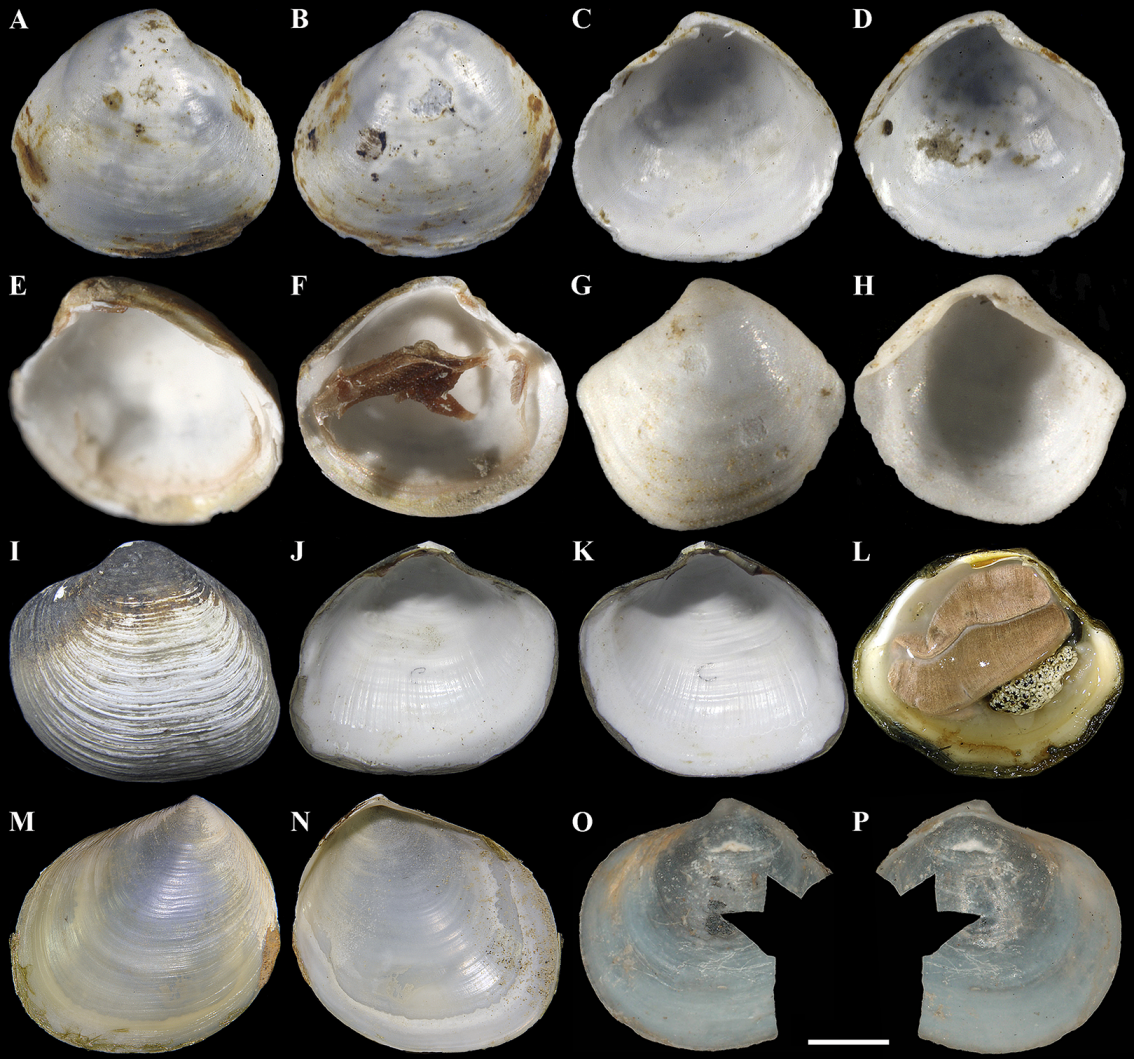
Species of the genus *Axinulus*. (A–D) *Axinulus careyi*, holotype (LACM 1900), shell length 2.6 mm. (E and F) *Axinulus careyi*, paratype (SBMNH 83295), shell length 2.2 mm. (G and H) *Axinulus thackergeigeri*, holotype (SBMNH 149742), shell length 1.9 mm. (I–K) *Axinulus hadalis*, paratype-c (NMNS Mo 71432-c), shell length 34.9 mm. (L) *Axinulus hadalis*, shell length 41.0 mm (photo by Dr. Y. Fujiwara (Japan Agency for Marine-Earth Science and Technology, Yokosuka, Japan)). (M and N) *Axinulus obliquus*, holotype (NSMT Mo 69719), shell length 19.0 mm. (O and P) *Cryptodon (Axinulus) simplex*, holotype (USNM 159888) (photo from the USNM Web site, creator: Barbara Valentas-Romera, “Information provided with the permission of the National Museum of Natural History, Smithsonian Institution, 10th and Constitution Ave. N.W., Washington, DC 20560-0193 (http://www.nmnh.si.edu/)”). Scale bars: O and P = 1 mm.

In *A. careyi*, the hinge plate of both valves bears relatively large cardinal tubercles (Figs. 2A–2F). According to shell proportions and hinge structure, this species, in actuality, more conforms to the genus *Mendicula* (Payne & Allen, 1991; Kamenev & Nadtochy, 2000; Oliver & Killeen, 2002; Zelaya, 2010). However, unlike *Mendicula*, it has a distinct, long and deep escutcheon (Valentich-Scott et al., 2014). Examination of the anatomy of *A. careyi* is needed for a more reliable generic assignment.

A very small-sized *A. thackergeigeri* has a shell that strongly differs from *Axinulus* in shape and proportions and a variably developed escutcheon on different valves (Figs. 2G and 2H), and its anatomy was not studied (Coan & Valentich-Scott, 2012). This species also requires further investigation and very probably should be referred to a different thyasirid genus.

*Axinulus hadalis* has a very large and thick shell (Figs. 2I–2L), its shell morphology strongly differs from that of the type of the genus *Axinulus*. In addition, the ctenidium of this species consists of two demibranchs. *Axinulus hadalis* was originally referred to the genus *Maorithyas* (Okutani, Fujikura & Kojima, 1999). However, after studying the type of *Maorithyas*, *Maorithyas marama* Fleming, 1950, Oliver with coauthors (Oliver & Sellanes, 2005; Oliver, 2014; Åström, Oliver & Carrolla, 2017) came to the conclusion that allocation of this species to *Maorithyas* is incorrect as both shell and anatomy are not in agreement. Valentich-Scott et al. (2014) also examined the shell morphology of holotypes of *M. marama* and *M. hadalis* and came to the conclusion that *M. hadalis* does not correspond to *Maorithyas* and perhaps belongs to a new genus of the Thyasiridae. Despite that its anatomy was not investigated, it was reported that the digestive system of *A. hadalis* is greatly reduced and the large and thick ctenidium harbors intracellular symbiotic bacteria (Fujikura et al., 1999; Okutani, Fujikura & Kojima, 1999; Fujiwara et al., 2001). This very interesting species was found in the Japan Trench at a depth of 7,326–7,434 m exceptionally in hydrothermal vent areas, which is currently the deepest record of a thyisirid harboring symbiotic bacteria (Fujikura et al., 1999; Fujiwara et al., 2001; Sasaki, Okutani & Fujikura, 2005). Without doubt, *A. hadalis* needs further study and reassignment to a different genus or placement in a new genus based on its morphological and anatomical characters.

*Axinulus obliquus* Okutani, 1968 was described as *A. obliqua* following only examination of the morphology of the shell (Okutani, 1968) (Figs. 2M and 2N). This species has a large shell (up to 19 mm) that is similar in its main morphological features to *Thyasira sensu stricto*. Most likely, the ctenidium of this species consists of two demibranchs and after further anatomical investigation *A. obliquus* can be placed in the genus *Thyasira*.

In addition, Huber (2015) assigned small-sized species, *Axinulus exintermedius* (Gaglini, 1992) and *Axinulus simplex* (Verrill & Bush, 1898), to the genus *Axinulus*. Judging from a photo given in Gaglini (1992), *A. exintermedius* has a shell very similar to *Axinulus* in shape and proportions; however, clarification of its taxonomic position requires further morphological and anatomical investigations. Verrill & Bush (1898) described *A. simplex* as *Cryptodon* (*Axinulus) simplex* Verrill & Bush, 1898 from a single imperfect specimen, which was found in the Atlantic Ocean (39°56′N, 69°45′W) at a depth of 638 m. The species description contains a figure only of the hinge of right valve and the interior of left valve of type specimen. One damaged left valve of the holotype is stored in the collection of the USNM (Figs. 2O and 2P). The shape and proportions of the shell of this species differ from those of *Axinulus*, and Dall (1901) suggested that it may even not belong to the Thyasiridae. *Axinulus simplex* is similar in shell shape and proportions to *Thyasira subcircularis* Payne & Allen, 1991. Obviously, further study is needed to clarify the taxonomic position of this species.

**Figure 3:**
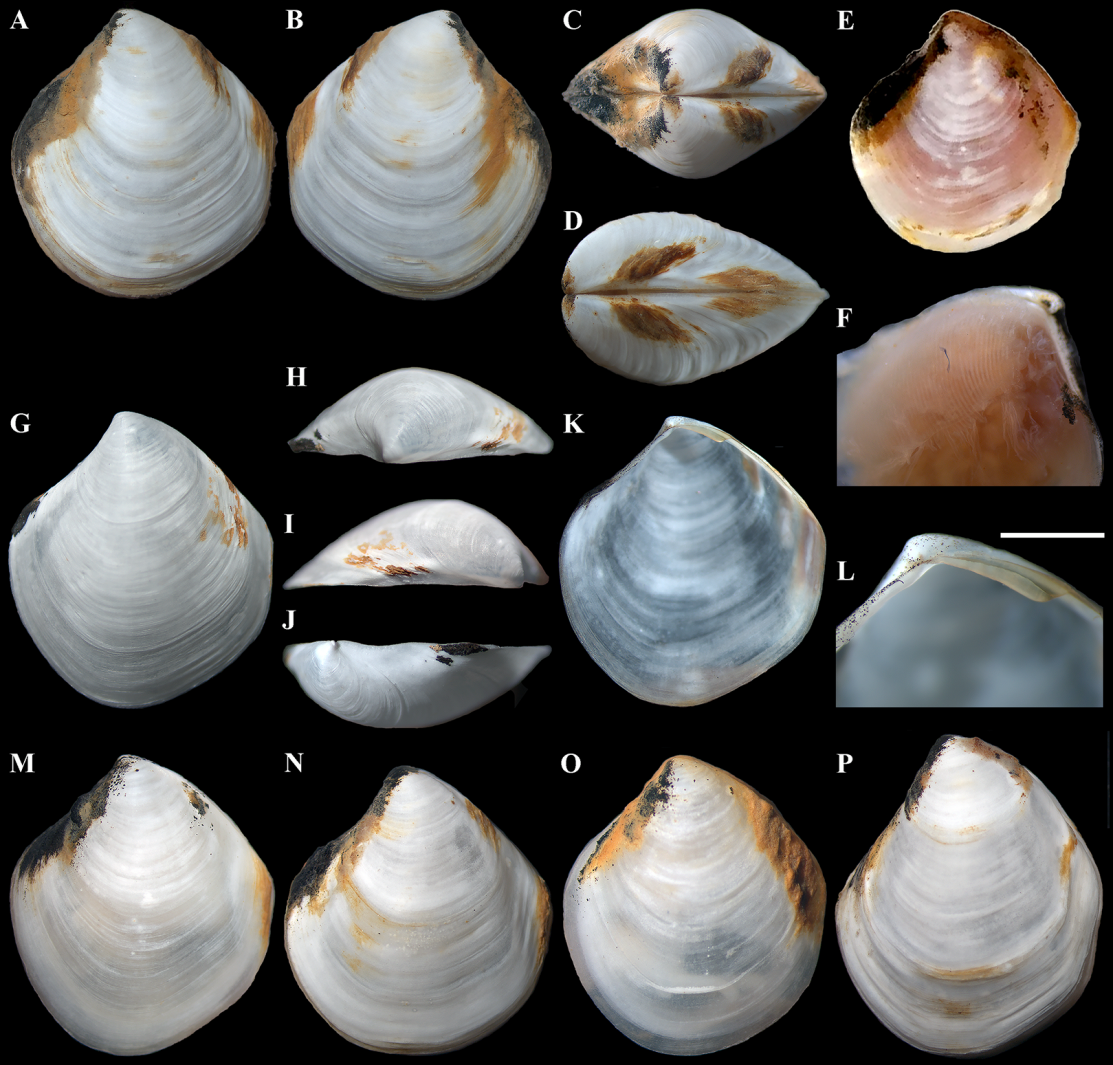
*“Axinulus” roseus* sp. nov. (A–D) Holotype (MIMB 40337), exterior, dorsal, and posterodorsal views of both valves, shell length 8.7 mm. (E) Live specimen, shell length 7.0 mm. (F) Body part of a living specimen, shell length 7.0 mm. (G–J) Exterior, dorsal, posterodorsal, and anterodorsal views of left valve cleaned of periostracum and deposit, valve length 7.2 mm. (K and L) Interior view and ligament of right valve, valve length 6.3 mm. (M–P) Variability of shell shape, exterior view of left valves: (M) Paratype (MIMB 40338), shell length 6.5 mm; (N) Paratype (MIMB 40338), shell length 8.0 mm; (O) Specimen from holotype locality, shell length 4.1 mm; (P) Specimen from holotype locality, shell length 7.1 mm. Scale bar: L = 1 mm.

**Figure 4:**
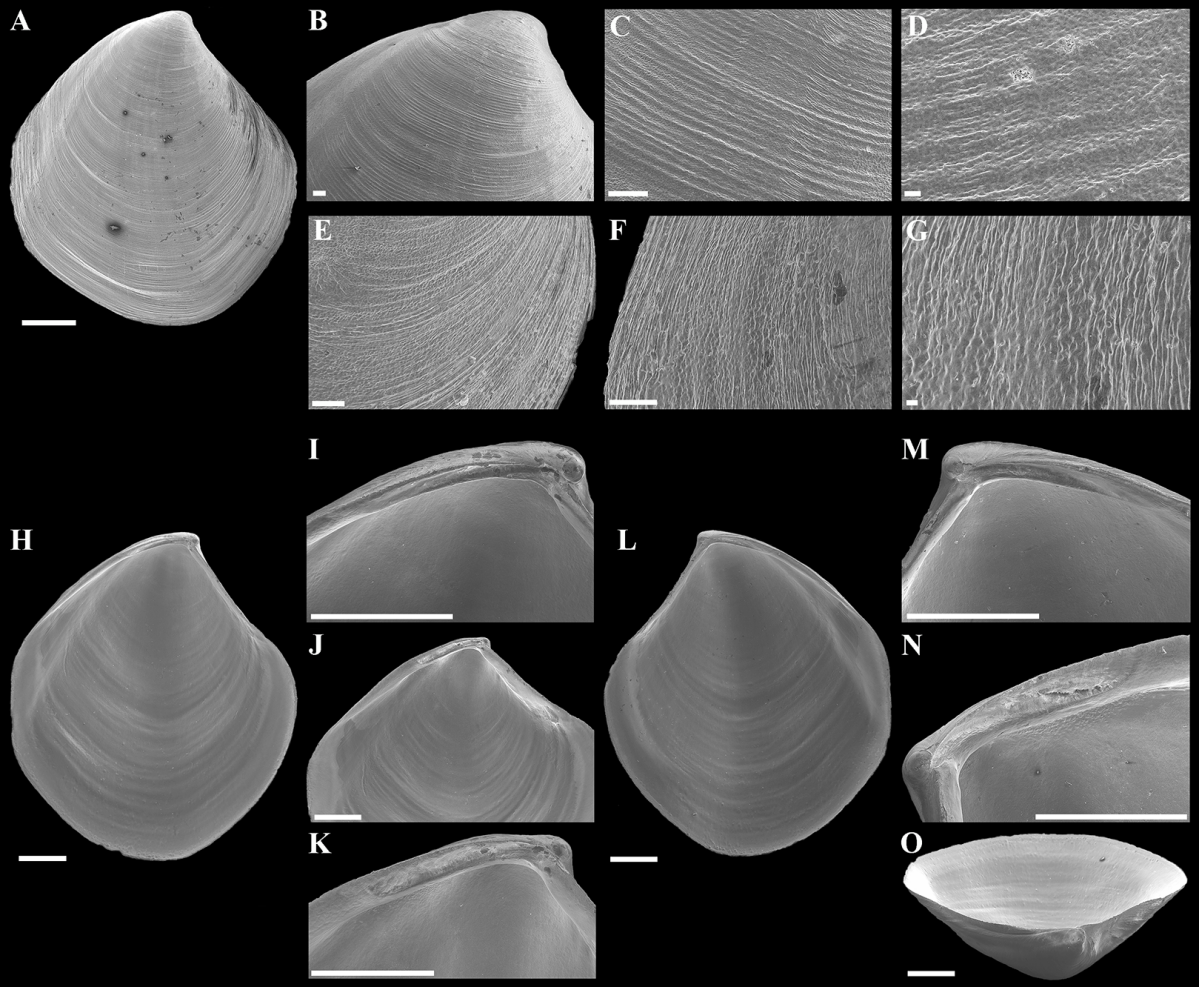
Scanning electron micrographs of *“Axinulus” roseus* sp. nov. (A) Exterior view of right valve. (B) Sculpture of dorsal region of shell. (C and D) Sculpture of central shell part. (E) Sculpture of anterior shell end. (F and G) Sculpture of posterior shell end. (H) Interior view of left valve. (I) Hinge plate and resilifer of left valve. (J and K) Ventral view of hinge plate and resilifer of left valve. (L) Interior view of right valve. (M) Hinge plate and resilifer of right valve. (N) Ventral view of resilifer of right valve. (O) Dorsal view of right valve. Scale bars: A and H–O = 1 mm; B, C, E and F = 100 µm; D and G = 10 µm.

**Figure 5:**
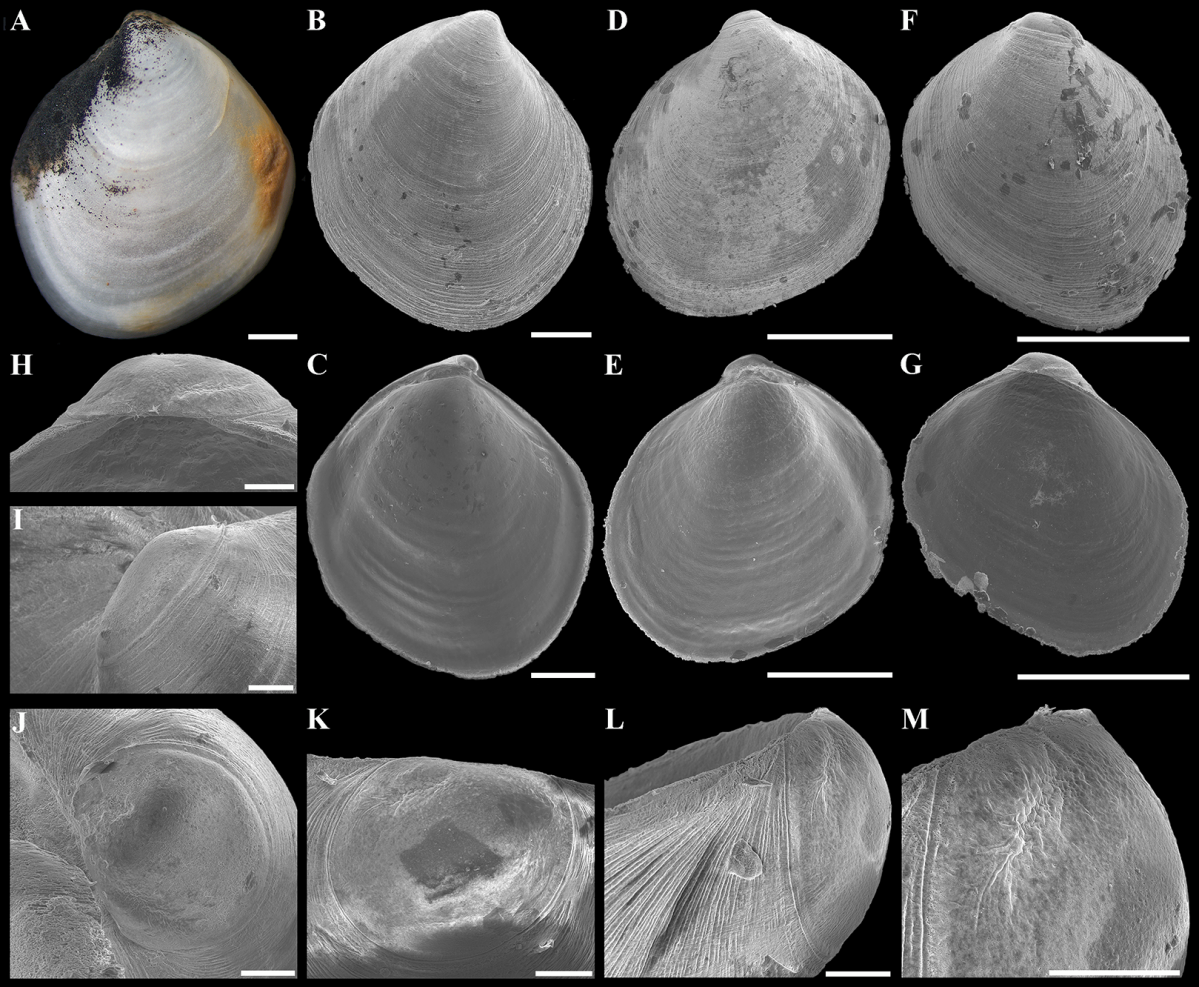
Shells of young specimens and prodissoconches of *“Axinulus” roseus* sp. nov. (A) Exterior view of left valve. (B–G) Scanning electron micrographs of shells of three young specimens: (B and C) Exterior view of right valve and interior view of left valve; (D and E) Exterior view of left valve and interior view of right valve; (F and G) Exterior view of right valve and interior view of left valve. (H–M) Scanning electron micrographs of prodissoconch of different specimens showing prodissoconch shape and sulcus position. Scale bars: A–G = 500 µm; H–M = 50 µm.

**Figure 6:**
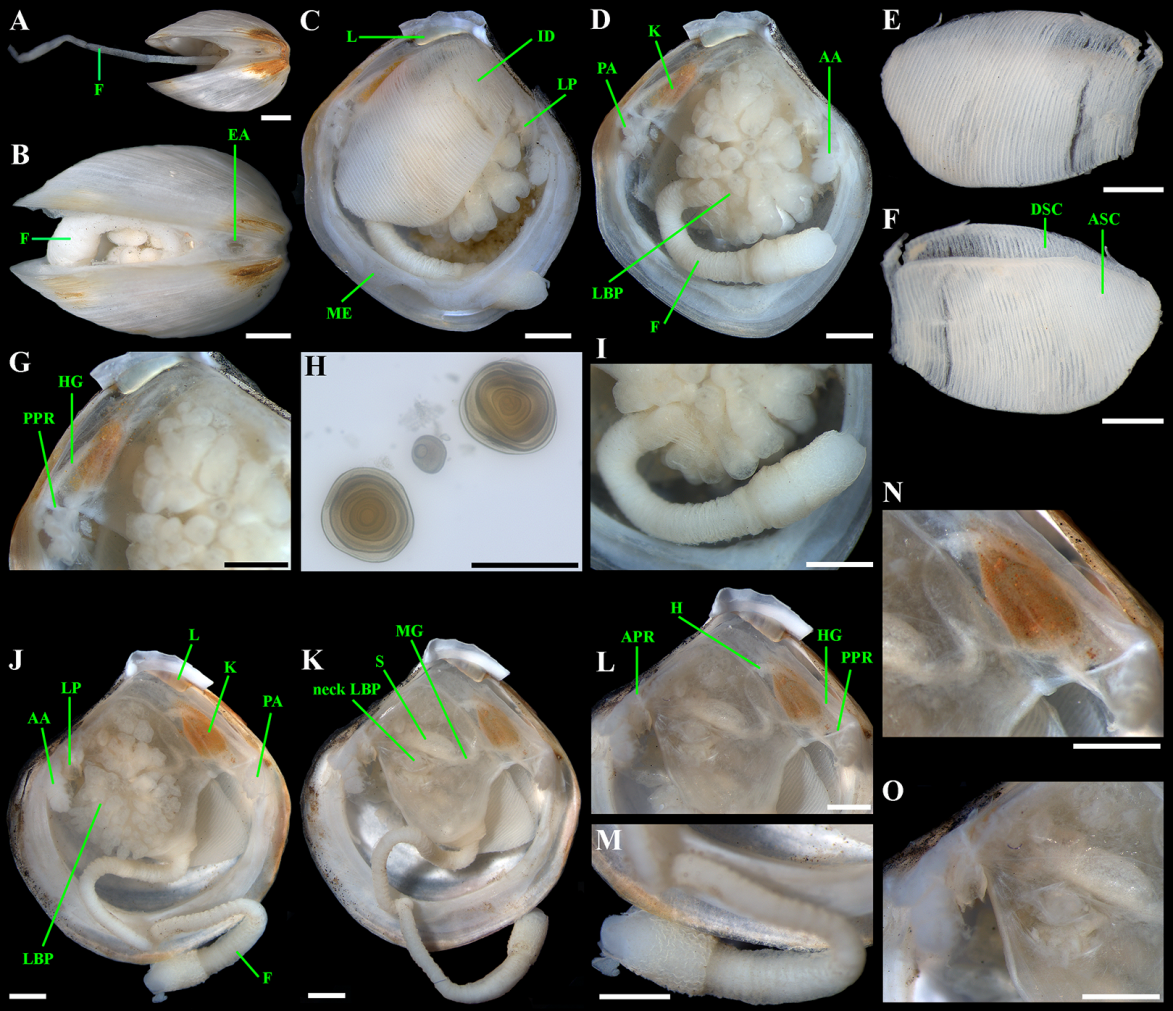
*“Axinulus” roseus* sp. nov. (A and B) Exhalant aperture. (C) Gross anatomy after removal of right valve and mantle. (D) Gross anatomy after further removal of right ctenidium. (E and F) Ctenidium. (G) Kidney with orange granules. (H) Kidney granules (photo by Dr. O.V. Yurchenko (NSCMB FEB RAS)). (I) Foot. (J) Gross anatomy after removal of left valve, mantle, and left ctenidium. (K) Gross anatomy after further removal of left lateral body pouch. (L) Digestive system. (M) Foot. (N) Kidney, midgut, and hindgut. (O) Labial palps and stomach. Abbreviations: A, anterior adductor muscle; APR, anterior pedal retractor muscle; ASC, ascending lamella; DSC, descending lamella; EA, exhalant aperture; F, foot; H, heart; HG, hind gut; ID, inner demibranch; K, kidney; L, ligament; LBP, lateral body pouch; LP, labial palps; ME, mantle edge; MG, mid gut; PA, posterior adductor muscle; PPR, posterior pedal retractor muscle; S, stomach. Scale bars: A–G and I–O = 1 mm; H = 100 µm.

**Figure 7:**
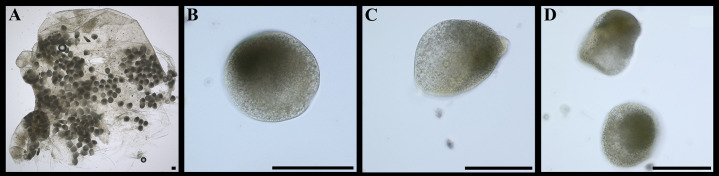
*“Axinulus” roseus* sp. nov. (A) Part of gonad containing eggs. (B–D) Eggs. Scale bars: A–D = 100 µm. Photos by Dr. O.V. Yurchenko (NSCMB FEB RAS).

**Figure 8:**
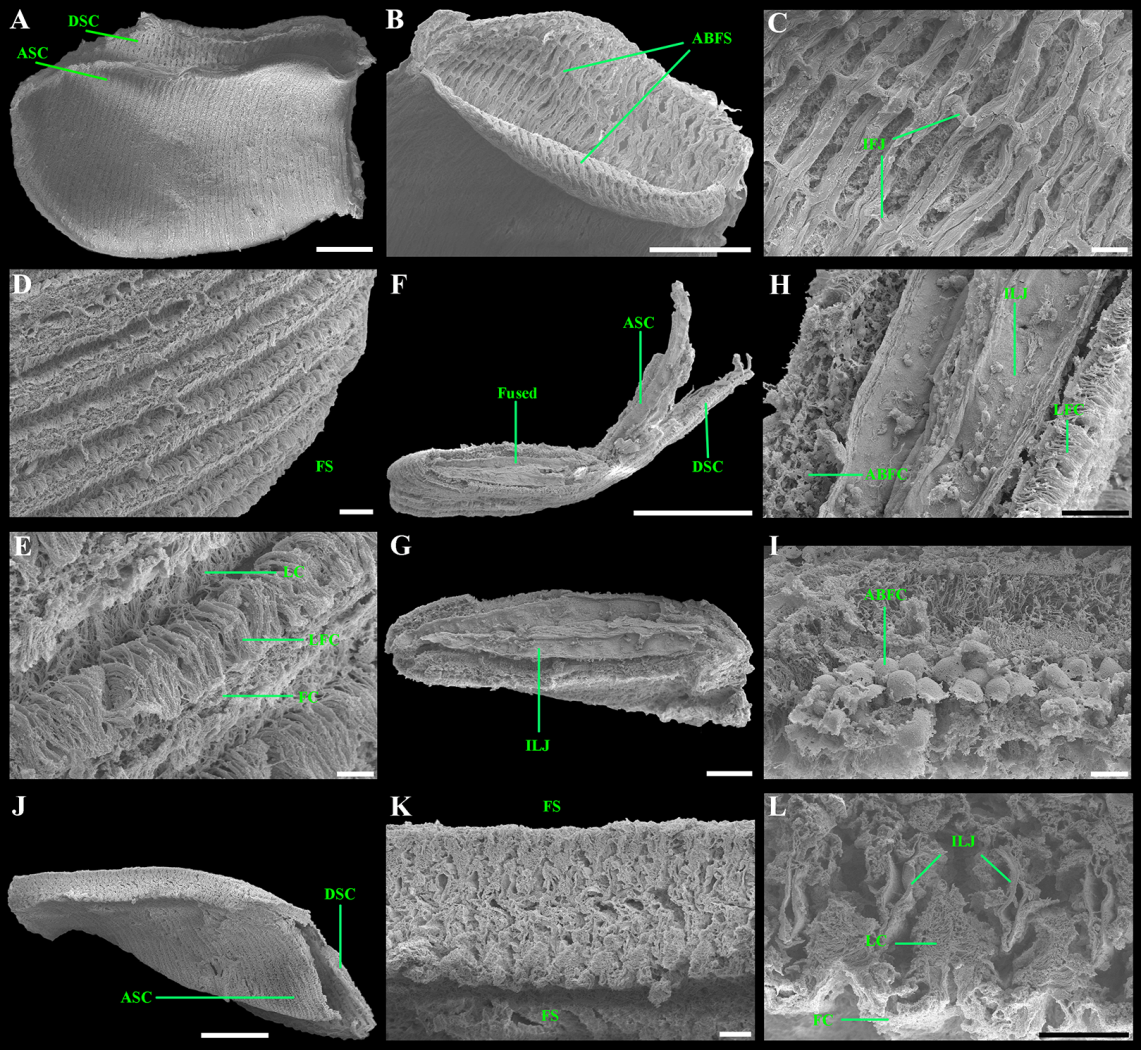
Scanning electron micrographs of the ctenidium and filaments of *“Axinulus” roseus* sp. nov. (A) Inner demibranch. (B and C) Abfrontal surface of filaments showing inter-filamentar junctions. (D) Frontal surface of descending lamellae. (E) Ciliaton of filaments. (F) Transverse section of inner demibranch. (G–I) Transverse section of ventral part of inner demibranch showing inter-lamellar junction and abfrontal cells. (J–L) Cross section of inner demibranch showing inter-lamellar junctions. Abbreviations: ABFC, abfrontal cell; ABFS, abfrontal surface; ASC, ascending lamella; DSC, descending lamella; FC, frontal cilia; FS, frontal surface; IFJ, inter-filamentar junction; ILJ, inter-lamellar junction; LC, lateral cilia; LFC, lateral frontal cirri. Scale bars: A, B, F and J = 500 µm; G = 100 µm; C, D, H, K and L = 50 µm; E and I = 10 µm.

**“*Axinulus*” *roseus* sp. nov.**

(Figs. 3–8, Tables 1 and 2)

**Table 1:** Additional material of “*Axinulus” roseus* sp. nov. examined in the present study.

Ship, cruise no.	Station	Date	Start		End		Depth (m)	Gear	*N*
			Latitude °N	Longitude °E	Latitude °N	Longitude °E			
Kuril-Kamchatka Trench									
*Vityaz* 14	2217	29.06.1953	44°07′	150°32′	–	–	9,050–9,000	ST	19
*Sonne* 250	67	10.09.2016	45°12.944′	152°42.844′	–	–	9,495	GKG	99
	77	13.09.2016	45°13.892′	152°50.774′	45°14.219′	152°49.956′	9,577–9,583	EBS	181
	79	14.09.2016	45°12.943′	152°42.821′	–	–	9,449	GKG	70
	100	20.09.2016	44°12.378′	150°39.053′	–	–	9,305	GKG	49
	102	20.09.2016	44°11.997′	150°33.766′	44°12.003′	150°32.745′	9,537–9,474	EBS	5
	103	21.09.2016	44°12.499′	150°39.055′	44°12.502′	150°37.258′	9,301–9,431	AGT	210
	105	22.09.2016	44°12.391′	150°36.006′			9,540	GKG	32

**Note:**

ST, Sigsbee trawl; GKG, giant box corer (0.25 m^2^); EBS, epibenthic sledge; AGT, Agassiz trawl; *N*, number of live specimens.

**Table 2:** *“Axinulus” roseus* sp. nov. Shell measurements (mm), indices and summary statistics of indices.

Depository	*L*	*H*	*A*	LL	EL	*W*	*H*/*L*	*A*/*L*	LL/*L*	EL/*L*	*W*/*L*
Holotype MIMB 40337	8.7	9.3	3.7	3.5	5.5	5.5	1.069	0.425	0.402	0.632	0.632
MIMB	8.5	9.2	3.5	3.8	5.0	5.5	1.082	0.412	0.447	0.588	0.647
Paratype MIMB 40338	6.5	7.3	2.7	3.0	3.5	4.0	1.123	0.415	0.462	0.538	0.615
Paratype MIMB 40338	8.1	9.0	3.1	3.0	5.8	5.3	1.111	0.383	0.370	0.716	0.654
Paratype MIMB 40338	7.7	8.7	3.0	3.5	5.2	5.0	1.130	0.390	0.455	0.675	0.649
Paratype MIMB 40338	8.0	8.8	3.1	4.0	5.2	5.0	1.100	0.388	0.500	0.650	0.625
MIMB	7.5	8.5	3.0	3.5	5.0	4.6	1.133	0.400	0.467	0.667	0.613
Paratype MIMB 40338	7.4	8.4	2.7	3.4	4.7	4.5	1.135	0.365	0.459	0.635	0.608
Paratype MIMB 40338	7.5	8.2	3.2	3.5	4.6	4.3	1.093	0.427	0.467	0.613	0.573
Paratype MIMB 40338	7.4	8.2	2.7	4.0	5.3	4.5	1.108	0.365	0.541	0.716	0.608
Paratype MIMB 40338	6.6	8.0	2.5	3.2	4.0	4.1	1.212	0.379	0.485	0.606	0.621
Paratype MIMB 40338	7.1	8.3	3.0	3.4	4.6	4.5	1.169	0.423	0.479	0.648	0.634
Paratype MIMB 40338	6.6	7.3	2.5	3.2	4.5	4.1	1.106	0.379	0.485	0.682	0.621
MIMB	7.1	8.4	2.2	3.6	4.7	4.3	1.183	0.310	0.507	0.662	0.606
MIMB	6.9	8.6	2.5	4.8	5.0	4.8	1.246	0.362	0.696	0.725	0.696
MIMB	6.0	7.0	2.2	2.9	4.0	3.6	1.167	0.367	0.483	0.667	0.600
MIMB	2.9	3.4	1.3	1.5	1.9	1.8	1.172	0.448	0.517	0.655	0.621
MIMB	4.1	4.7	2.0	1.9	2.4	2.6	1.146	0.488	0.463	0.585	0.634
MIMB	2.7	2.9	1.0	1.3	1.5	1.6	1.074	0.370	0.481	0.556	0.593
MIMB	3.0	3.6	1.0	1.6	1.9	2.0	1.200	0.333	0.533	0.633	0.667
MIMB	2.9	3.1	1.1	1.4	1.9	1.9	1.069	0.379	0.483	0.655	0.655
MIMB	2.3	2.6	0.9	1.0	1.6	1.2	1.130	0.391	0.435	0.696	0.522
MIMB	2.2	2.6	0.8	1.0	1.5	1.2	1.182	0.364	0.455	0.682	0.545
MIMB	3.7	4.4	1.4	1.6	2.5	2.3	1.189	0.378	0.432	0.676	0.622
MIMB	4.2	5.0	1.5	1.8	2.7	2.6	1.190	0.357	0.429	0.643	0.619
Statistics	*L*	*H*	*A*	LL	EL	*W*	*H*/*L*	*A*/*L*	LL/*L*	EL/*L*	*W*/*L*
Mean	–	–	–	–	–	–	1.141	0.388	0.477	0.648	0.619
SE	–	–	–	–	–	–	0.041	0.027	0.037	0.036	0.025
SD	–	–	–	–	–	–	0.048	0.037	0.059	0.047	0.036
Min	–	–	–	–	–	–	1.069	0.310	0.370	0.538	0.522
Max	–	–	–	–	–	–	1.246	0.488	0.696	0.725	0.696

**Note:**

*L*, shell length; *H*, height; *W*, width; *A*, anterior end length; LL, lunule length; EL, escutcheon length.

*Axinulus* sp. 1: Kamenev, 2019, p. 6, 7.

urn:lsid:zoobank.org:act:28594E9C-C258-4305-A75F-DD2066FDA885

**Type material and locality:** Holotype (MIMB 40337), Kuril-Kamchatka Trench, Pacific Ocean (44°12.499′N, 150°39.055′E–44°12.502′N, 150°37.258′E), 9,301–9,431 m, Agassiz trawl, Coll. K.V. Minin, 21-IX-2016 (RV *Sonne*, cruise no. 250, stn. 103); paratypes (10) (MIMB 40338), from holotype locality; paratypes (10) (SMF (Senckenberg Museum Frankfurt) 360675) from holotype locality.

**Other material examined:** 665 live specimens (Table 1).

**Diagnosis:** Shell relatively large (to 8.7 mm in length), rhomboidal, subequilateral, slightly drawn out anteriorly. Sculpture of narrow, commarginal ridges forming weak, commarginal undulations. Shell surface with pitted micro-sculpture. Anterodorsal shell margin slightly concave. Posterodorsal shell margin slightly convex. First posterior fold absent. Second posterior fold very weak. Posterior sulcus very weak. Submarginal sulcus long, sharply defining an escutcheon. Escutcheon very long, narrow, deep. Auricle absent. Lunule long, wide, shallowly sunken. Ligament internal, partially visible externally. Prodissoconch large (to 225 µm), slightly drawn out anteriorly, irregularly convex, flattened anteriorly; anterior end of prodissoconch bearing an oblique, elongated sulcus with numerous fine, curved folds. Lateral body pouches large, with numerous small projecting lobes.

**Description.** Shell relatively large (to 8.7 mm in length and 9.3 mm in height), rhomboidal, equivalve, subequilateral, white, thin, fragile, translucent, strongly inflated (W/L = 0.619 ± 0.025), slightly shorter than high (H/L = 1.141 ± 0.041), slightly drawn out anteriorly; median area divided by a weak change in angulation; patches of ferruginous and silty deposit adhering to anterodorsal and posterodorsal shell margins (Fig. 3; Table 2). Periostracum very thin, colorless, translucent, adherent. Sculpture of conspicuous, closely spaced, narrow, commarginal ridges with sparse, very fine, thin, radial rays from microscopic concentric wrinkles forming weak, wider, irregular, commarginal undulations; commarginal ridges more closely spaced at shell margins. Shell surface with pitted micro-sculpture of very small (to 5 µm), shallow, densely spaced pits, forming small tubercles on shell margins. Beaks small, raised, prosogyrate, slightly anterior to midline (A/L = 0.388 ± 0.027) (Fig. 4; Table 2). Anterodorsal shell margin slightly concave, straight in young specimens, sloping steeply from beaks, forming a rounded angle at transition to anterior margin. Anterior shell margin slightly curved, smoothly transitioning to ventral margin. Ventral margin strongly curved, slightly angulate at ventral extremity. Posterodorsal shell margin slightly convex or almost straight, sloping steeply from beaks, forming a distinct angle at transition to posterior margin. First posterior fold absent. Second posterior fold very weak and strongly rounded. Posterior sulcus very weak and shallow, indistinct. Submarginal sulcus long, deeply incised with almost vertical margins, sharply defining an escutcheon. Escutcheon very long (EL/L = 0.648 ± 0.036), narrow, deep, flattened. Auricle absent. Lunule long (LL/L = 0.477 ± 0.037), wide, shallowly sunken, well expressed along entire anterodorsal shell margin, demarcated by rounded, weak ridges extending along anterodorsal shell margin from beaks to anterior shell margin. Ligament opisthodetic, internal, partially visible externally, thick, evenly curved, relatively short, about one-third the length of escutcheon, lying in shallow, slightly curved, narrow resilifer. Prodissoconch large (length 212–225 µm), distinct, sharply separated from shell, ovate in outline, slightly drawn out anteriorly, irregularly convex, flattened anteriorly. Anterior end of prodissoconch bearing an oblique, shallow, wide, elongated sulcus with numerous fine, curved folds of variable length; remaining surface of prodissoconch with densely spaced, very shallow pits. (Fig. 5). Hinge plate thin, edentulous, with a small swelling beneath beak in each valve (Figs. 4I and 4M). Interior of valves white, muscle scars indistinct.

*Anatomy:* Mantle thin, margins thickened. Mantle fusion limited to a small inter-connection below the posterior adductor, forming an exhalant aperture. Anterior adductor muscle elongated, curved almost parallel to anterior shell margin. Posterior adductor muscle 2 times shorter than anterior, ovate. Foot very long, vermiform, with bulbous distal section; surface of bulbous portion with densely spaced papillae. Heel obsolete. Anterior and posterior pedal retractors short, not strongly developed (Fig. 6).

Labial palps small (to 1 mm in length), narrow, triangular. Alimentary system with short oesoghagus leading to a relatively large, elongate stomach; combined style sac and midgut strongly curved; hind gut forming an anterior loop dorsal to style sac, passing through the heart and running posteriorly dorsal to kidney and posterior adductor muscle, opening at ventral side of posterior adductor muscle (Figs. 6J–6O). Lateral pouches bright pink in live specimens (Fig. 3F), large, with numerous small projecting lobes; finger-like terminations thick, cloven or single; each pouch connecting to body by a narrow neck. Kidneys large, dorsoventrally elongated, occupying a posterodorsal position between posterior adductor muscle and heart, containing numerous, bright orange, small (to 80 µm in diameter), different-size granules (Figs. 6G, 6H, 6J–6L and 6N). Gonad occupying inner side of lateral pouches. Sexes are separate. Eggs oval or polygonal (up to 145 µm in length after fixation) (Fig. 7).

Ctenidium thin, wide, consisting of a single inner demibranch with fully reflected filaments (up to 60 filaments in large specimens). Demibranchs covering greater part of lateral body pouches. Demibranch consisting of both ascending and descending lamellae; ascending lamellae near four fifths of descending lamellae length; ascending and descending lamellae fused over half of their length (Figs. 6E, 6F, 8A and 8F)). Adjacent filaments joined by fine, muscular inter-filamentar junctions at about every 125–150 µm (Figs. 8B and 8C). Filaments narrow with abfrontal tissue not extended or thickened (Figs. 8J and 8K). Filaments of asceding and descending lamellae joined in ventral part of ctenidium by inter-lamellar junctions (Figs. 8F–8H and 8L). Frontal surfaces of filaments ciliated with short frontal cilia, long lateral frontal cirri and below these long lateral cilia (Figs. 8D and 8E). Bacteriocytes absent. Abfrontal cells resembling bacteriocyte, not harboring endosymbiotic bacteria (Figs. 8H and 8I).

**Variability:** Shell shape and proportions distinctly change with age (Figs. 5A–5G; Table 2). In young specimens (up to 3 mm in shell length), in contrast to adults, the shell is relatively higher, less angulate, with a strongly curved and anteriorly drawn-out ventral margin; anterodorsal and posterodorsal margins are smoothly curved or straight; the lunule is less expressed; the posterior sulcus is barely visible; commarginal undulations are less distinct or lacking. In adult specimens, the shell shape and proportions also vary significantly (Figs. 3M–3P). The shell height and width, the length of the lunule and escutcheon, the degree of curving of shell margins, the depth of the posterior sulcus vary. Some specimens have a shell rather elongated dorsoventrally. In some specimens, the anterodorsal margin is more concave and the posterodorsal margin is more convex, the ventral margin is more curved and more drawn out anteriorly, posterior sulcus is almost invisible.

**Figure 9:**
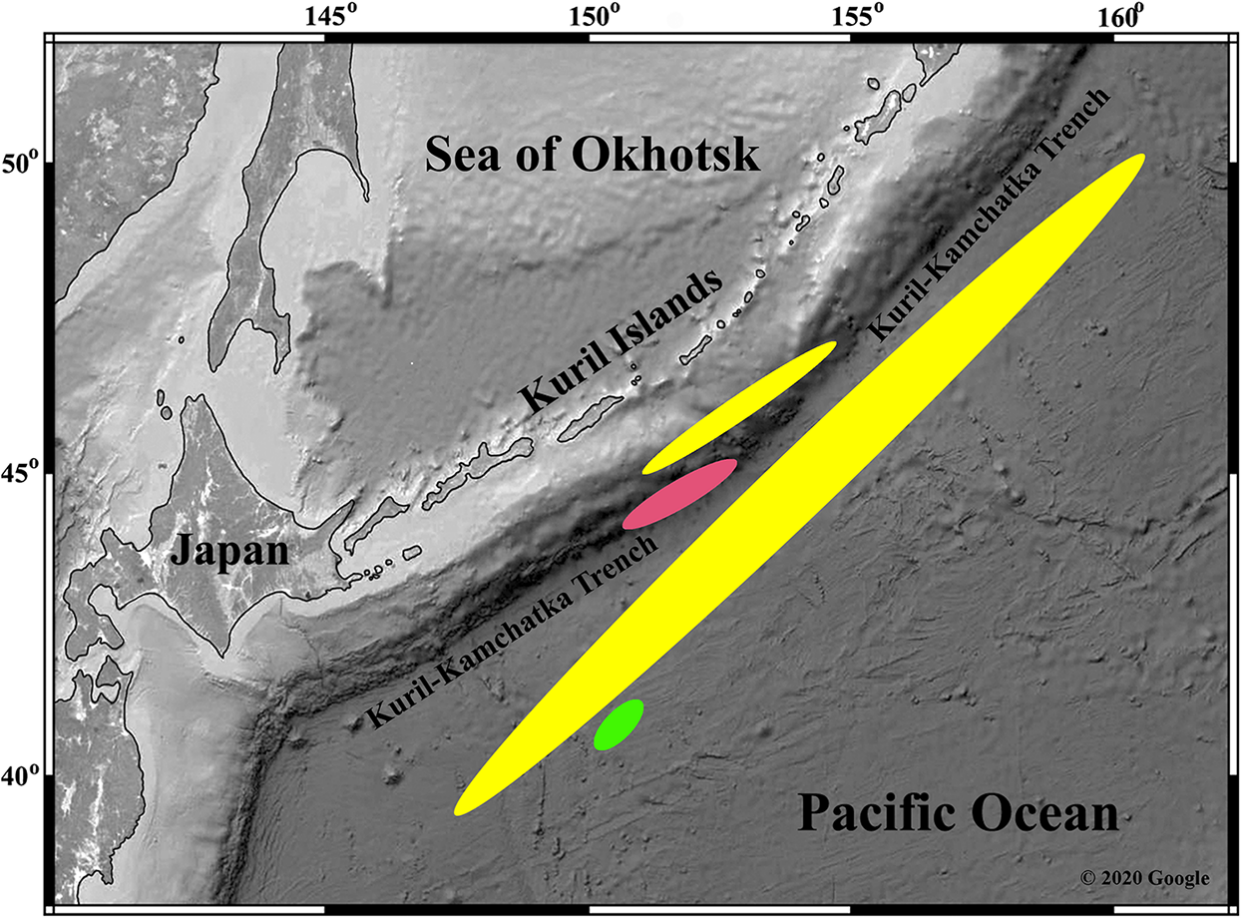
Distribution of the described species of Thyasiridae. *“Axinulus” roseus* sp. nov. (pink area); *“Axinulus” oliveri* sp. nov. (yellow area); *Parathyasira fragilis* sp. nov. (green area). (Map data © 2020 Google).

**Distribution:** This species was recorded on the bottom of the deepest basin of the Kuril-Kamchatka Trench (44°07′N, 150°32′E–45°14.219′N, 152°49.956′E) at 9,000–9,583 m depth (Fig. 9).

**Comparisons:** *“Axinulus” roseus* sp. nov. strongly differs from all species of the genus *Axinulus* in having a large, angulate, rhomboidal and dorsoventrally elongated shell with long and steeply sloping anterodorsal and posterodorsal shell margins, a projecting beak, and strong commarginal sculpture; a well expressed long, wide, excavated lunule demarcated by rounded ridges; a long and deep escutcheon; a large prodissoconch; and extensively lobed lateral pouches (Table 3). The new species is most similar in shell shape and proportions to *A. brevis*, but differs from it in having a large shell, a concave anterodorsal shell margin, well defined and deep lunule and escutcheon, distinct commarginal sculpture, a large prodissoconch (in *A. brevis* the prodissoconch is 135–175 µm in length), and extensively lobbed lateral pouches (Verrill & Bush, 1898; Payne & Allen, 1991; Oliver, 2015).

**Table 3:** Main differentiating characters of *Axinulus* species.

Species	Maximum shell length and height (mm)	Shell	Sculpture	Escutcheon	Auricle	Lunule	Ligament	Lateral pouch	Prodisso-conch length (µm)	References
*“Axinulus” roseus* sp. nov.	*L* = 8.7 *H* = 9.3	Rhomboidal; anterodorsal shell margin concave; posterodorsal shell margin convex or straight	Conspicuous, commarginal, narrow ridges with sparse, very thin, radial rays from microscopic concentric wrinkles and weak, irregular undulations; microsculpture of densely spaced pits	Well defined, very long, narrow	Absent	Well defined, sunken, long, wide	Visible externally	Large, extensively lobed	212–215	Present study
*“Axinulus” oliveri* sp. nov.	*L* = 5.7 *H* = 6.2	Ovate-rhomboidal; anterodorsal shell margin convex or straight; posterodorsal shell margin convex	Thin, commarginal ribs and weak, irregular undulations; microsculpture of densely spaced pits	Well defined, very long, narrow	Present, distinct	Weakly defined, with a crest, long, wide	Not visible externally	Large, extensively lobed	191–220	Present study
*Axinulus antarcticus*	*H* = 2.9	Subquadrate; anterodorsal shell margin horizontal, straight; posterodorsal shell margin straight	Thin, commarginal ribs	Absent	Present, distinct	Weakly defined, short, wide	Visible externally	Small, no marked lobes	115	Zelaya (2010)
*Axinulus philippinensis*	*L* = 3.2	Oblique, angular; anterodorsal shell margin convex or straight; posterodorsal shell margin convex or straight	Fine, growth lines and marginal, radial lines	Absent	Absent	No data	No data	Markedly lobed at margins	No data	Allen (2015)
*Axinulus alleni*	*L* = 2.5	Subquadrate; anterodorsal shell margin straight or convex; posterodorsal shell margin convex	Very fine, growth lines	Well defined, long, narrow	Present, distinct	No data	Visible externally	Large, swollen, without lobes	No data	Carrozza (1981) and Payne & Allen (1991)
*Axinulus brevis*	*L* = 2.70 *H* = 3.19	Upright oval outline; anterodorsal and posterodorsal shell margin convex	Very fine, growth lines	Absent	Absent	Weakly defined	Visible externally	Large, elongate, no marked lobes	No data	Verrill & Bush (1898) and Payne & Allen (1991)
*Axinulus croulensis*	*L* = 1.8 *H* = 2.0	Upright oval or almost circular outline; anterodorsal shell margin horizontal, straight; posterodorsal shell margin convex	Very fine, commarginal lines and growth stops, mostly glossy with a radial texture	Absent	Indistinct	Absent	Visible externally	Large, with small projecting lobes along dorsal margin	121–141	Payne & Allen (1991) and Oliver & Killeen (2002)
*Axinulus subequatorius*	*L* = 3.3 *H* = 3.5	Pyriform; anterodorsal and posterodorsal shell margins convex	Fine, ill-defined growth lines	Present	Absent	No data	No data	With a number of small peripheral lobes	No data	Payne & Allen (1991)

**Note:**

*L*, shell length; *H*, shell height.

**Derivation of name:** The specific name “roseus” (adjective in the nominative singular) refers to a bright pink color of large lateral pouches that shone through a translucent shell in all specimens (Figs. 3E and 3F).

**Remarks:** The new species is only provisionally placed in the genus *Axinulus*, as it differs from *A. brevis*, the type species of the genus *Axinulus*, in many respects. According to the size, shape, and proportions of the shell, as well as a number of morphological and anatomical features, the new species corresponds more to *Parathyasira*. The shared features include a rhomboidal shell, with a narrow and distinctly projecting umbo; the absence of first posterior fold and a very weak second posterior fold; the lack of the posterior sinus; a long deeply incised submarginal sulcus with almost vertical margins; a concave anterodorsal margin; an excavated lunule; a long and deep escutcheon; the absence of the auricle; a deeply sunken ligament; a weak hinge; large and extensively lobed lateral pouches (Payne & Allen, 1991; Oliver & Killeen, 2002; Rodrigues, Oliver & Cunha, 2008; Zelaya, 2009; Oliver, 2015). Many of these morphological and anatomical features are also characteristic for the species of the genus *Thyasira*. However, in contrast to *Parathyasira* and *Thyasira*, the new species has a ctenidium consisting of only one demibranch. This is an important diagnostic character used in the systematics of the family Thyasiridae. Five genera have a single demibranch: *Axinulus, Leptaxinus, Genaxinus, Mendicula*, and *Adontorhina*. All the genera include exclusively small-sized species with a shell length not greater than 5 mm and well differ in the external and internal morphology of shell, hinge structure, and anatomy (Scott, 1986; Payne & Allen, 1991; Kamenev, 1996, 2013; Coan, Scott & Bernard, 2000; Kamenev & Nadtochy, 2000; Oliver & Killeen, 2002; Oliver & Levin, 2006; Zelaya, 2010; Coan & Valentich-Scott, 2012). The shell of the new species is unusually large for species having a single demibranch. A comparative analysis of “*A.” roseus* sp. nov. and all type species of the genera having a single demibranch showed that, according to shell morphology and gill structure, it is most similar to the type of the genus *Axinulus. Axinulus brevis*, like *“A.” roseus* sp. nov., has an angular shell and elongate gill filaments with well developed filamentar muscle, skeletal rods, ciliation, as well as less developed abfrontal tissue than in larger species of *Thyasira* (Payne & Allen, 1991). However, as was noted above, *“A.” roseus* sp. nov. is distinguished from all species of the genus *Axinulus* by having a markedly larger shell with strongly projecting umbo, unusually long posterodorsal and anterodorsal margins, long, deep and distinct lunule and escutcheon, and extensively lobed lateral pouches. Most species of *Axinulus* have a shell with low beaks and indistinct lunule and escutcheon, lateral body pouches being simple, without or with a few indistinct lobes. Nevertheless, in contrast to other species of *Axinulus*, *A. alleni* and *A. subequatorius* have a long distinct escutcheon (Carrozza, 1981; Payne & Allen, 1991), while *A. subequatorius* has lobed lateral pouches. The lobed lateral pouches of *A. subequatories* were one of the features that did not allow Payne & Allen (1991) to place this species in the genus *Axinulus*. Furthermore, *A. croulinensis* has pouches with small projecting lobes along the dorsal margin (Payne & Allen, 1991). Judging from a figure provided in the species description (Allen, 2015), *A. philippinensis* also appears to possess pouches with projecting lobes. In addition, *A. antarcticus* and *A. alleni* have a distinct auricle. Thus, species of *Axinulus* exhibit a significant variation of shape of both the shell and lateral pouches. Nevertheless, I think that “*A.” roseus* sp. nov., is fairly unusual due to a combination of morphological and anatomical features; and therefore, I only provisionally place it in the genus *Axinulus*.

*“Axinulus” roseus* sp. nov. was found by the KuramBio II expedition in the Kuril-Kamchatka Trench in its deepest basin at a depth below 9,000 m only. At depths of 8,400–8,740 m, where the KuramBio II collected many samples, this species was no longer found (Kamenev, 2019). Among the material collected by *Vityaz* expeditions in the Kuril-Kamchatka Trench, this species was also found only in the sample collected deeper than 9,000 m. Probably, *“A.” roseus* sp. nov. occurs in the Kuril-Kamchatka Trench exceptionally at the maximum depths. It is possible that this species is endemic to the Kuril-Kamchatka Trench. I did not find this species in the samples collected by *Vityaz* expeditions in the Japan and Aleutian Trenches. These trenches are much shallower than the Kuril-Kamchatka Trench and their depth does not exceed 8,500 m. Although it is not ruled out that *“A.” roseus* sp. nov. can be found in the deepest part of the Japan Trench with depths of more than 8,400 m (Belyaev, 1989). The bottom fauna of the deepest part of the Japan Trench has not yet been studied. The *Vityaz* expeditions collected samples of macrobenthos in the Japan Trench only to 7,587 m depth (Belyaev, 1989).

*“Axinulus” roseus* sp. nov. forms extensive populations with high density in the deepest basin of the Kuril-Kamchatka Trench. The KuramBio II expedition collected 7 samples with various gear at a depth of more than 9,000 m, and *“A.” roseus* sp. nov. was found in all samples in large numbers (Table 1). The population density at a depth of about 9,500 m reached 396 ind. m^−2^, a high value of abundance even for shallow-water bivalve populations. Along with *Vesicomya sergeevi* Filatova, 1971 and *Parayoldiella ultraabyssalis* (Filatova, 1971), this species is the dominant among macrobenthos of the deepest basin of the Kuril-Kamchatka Trench (Kamenev, 2019).

Interestingly, only one of the seven samples collected at depths greater than 9,000 m contained large specimens of *“A.” roseus* sp. nov. This sample was collected with a trawl that sank deep into sediments and brought up a very large amount of silt. Probably, this large-sized species of thyasirid buries itself deep in the bottom sediments of the trench and is difficult to collect with other sampling gear. For example, live specimens of a large thyasirid *T. kaireiae*, with a shell length of up to 10 cm, that were collected using a ROV in the Japan Trench at a depth of more than 5,000 m, can bury in bottom sediments to a depth of more than 20 cm (Okutani & Fujikura, 2002). Smaller specimens of this species with a shell length of 4–5 cm were collected at about 10–20 cm depth of sediment (Fujikura et al., 2002). During the KuramBio II expedition, only the upper 20-cm layer of sediments from box-corer samples was thoroughly washed through a series of sieves, supposing that at such great depths of the Kuril-Kamchatka Trench bottom animals do not bury themselves in sediments to a depth of more than 20 cm. Perhaps, therefore, only relatively small specimens of *“A.” roseus* sp. nov. were found in the box-corer samples. It is also likely that large individuals are rarer or are distributed more aggregately, compared to small ones, and therefore, escaped the box-corer, which captures sediments from a small bottom area.

Juveniles of this species differ greatly from adults in shell morphology and anatomy and correspond much more to the diagnostic characters of the genus *Axinulus*. I had no doubt that the numerous juveniles were adults of a new species of the genus *Axinulus* until a sample with large specimens was collected at the end of the expedition. A similar case occurred with another thyasirid found at all depths of the Kuril-Kamchatka Trench. This species was collected in large numbers from various depths of the trench, only small individuals were found in many samples, some of which being already sexually mature. There was only one large individual and it changed my idea of what the morphology and true size of this species are. In this regard, it is possible that *A. philippinensis* from the Philippine Trench also has a larger shell and was described from juvenile individuals. This species was found only in three of 16 successful boxcorer samples collected in the Philippine Trench at 9,600–9,807 m depth (Allen, 2015). It was not found in epibenthic sledge samples. *A. philippinensis*, like *“A.” roseus* sp. nov., is characterized by significant variability in the shape and proportions of the shell, depending on its size. This species, like *“A.” roseus* sp. nov., has extensively lobbed pouches (Allen, 2015). Taking into account that the species richness and abundance of the benthic fauna in the Philippine Trench at maximum depths is much lower than in the Kuril-Kamchatka Trench (Belyaev, 1989; Jamieson, 2015), adults of *A. philippinensis* may be distributed much more sparsely than juveniles on the bottom of the trench, and could not be taken with a boxcorer because of its small capture area.

*Vityaz* expeditions collected successful trawl samples from the maximum depths of the Philippine Trench where bivalve mollusks were found (Belyaev & Mironov, 1977; Belyaev, 1989). No thyasirids were found in the samples after preliminary sorting. It is possible that final study of the collections of these expeditions will reveal the occurrence of thyasirids, including larger individuals of *A. philippinensis*.

**Figure 10:**
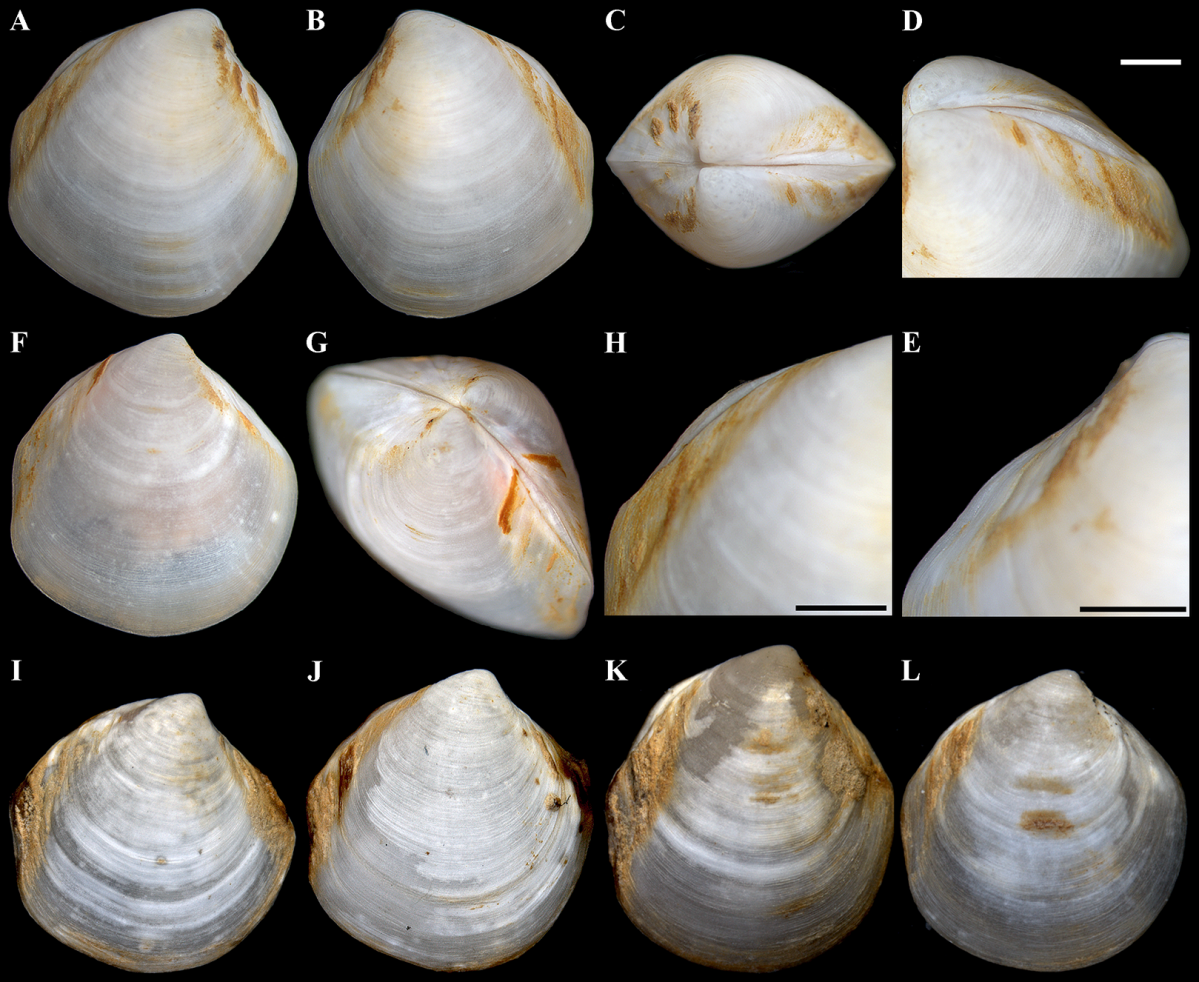
*“Axinulus” oliveri* sp. nov. (A–E) Holotype (MIMB 40339), exterior and dorsal view of both valves, oblique view of posterodorsal margin, and lateral view of anterodorsal margin of left valve showing lunule, escutcheon and auricle, shell length 5.6 mm. (F–H) Paratype (MIMB 40340), exterior view of right valve, oblique dorsal view of both valves, and lateral view of posterodorsal margin of right valve showing lunule and escutcheon, shell length 4.7 mm. (I–L) Variability of shell shape: I, shell length 3.9 mm; J, shell length 4.4 mm; K, shell length 4.6 mm; L, shell length 3.5 mm. Scale bars: D, E and H = 1 mm.

**Figure 11:**
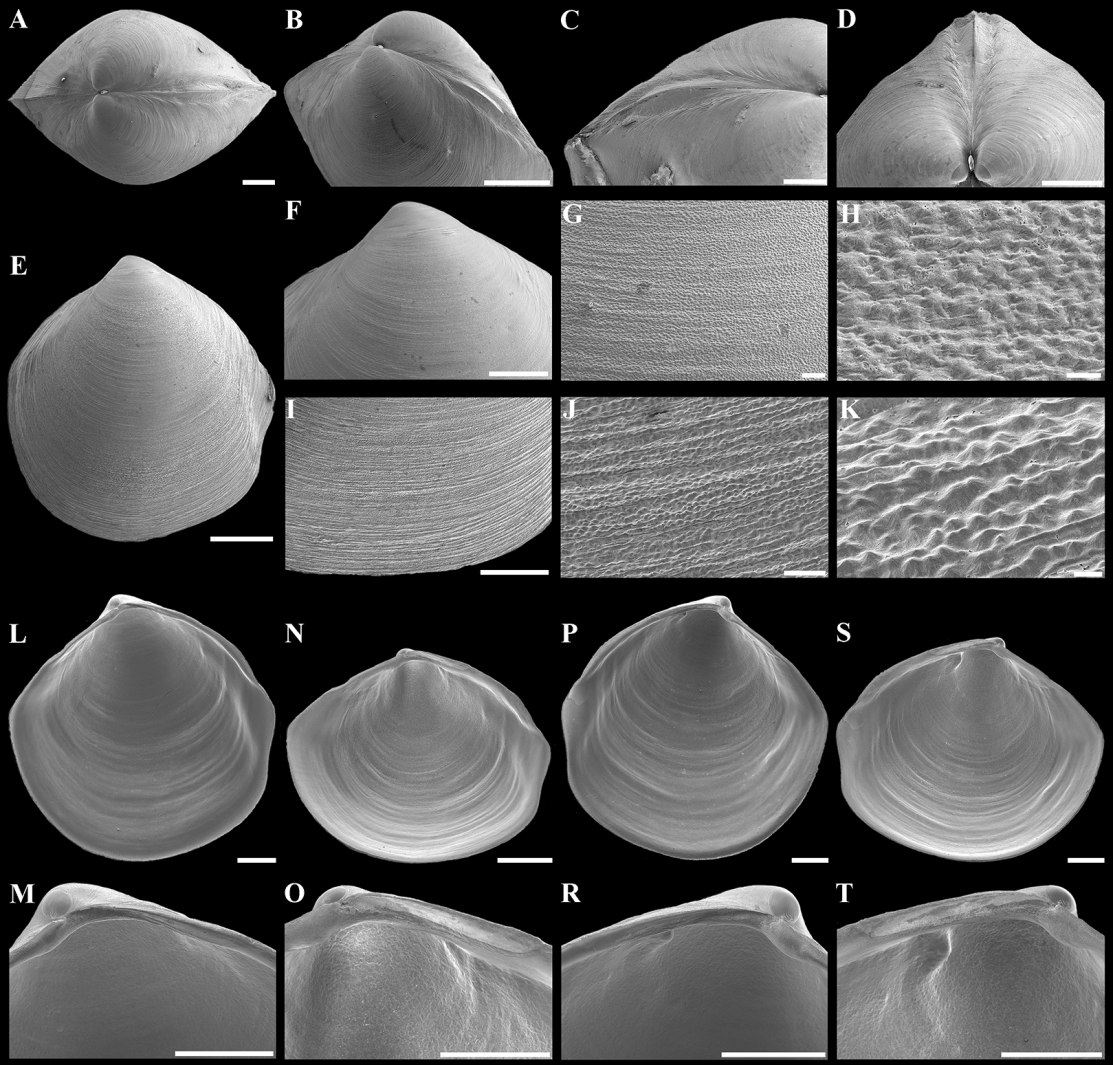
Scanning electron micrographs of *“Axinulus” oliveri* sp. nov. (A–D) Dorsal and oblique dorsal views of both valves, oblique and dorsal views of posterodorsal margin showing lunule, escutcheon, and auricle. (E) Exterior view of left valve. (F–H) Sculpture of dorsal region of shell. (I–K) Sculpture of ventral shell part. (L) Interior view of right valve. (M) Hinge plate and resilifer of right valve. (N and O) Ventral view of hinge plate and resilifer of right valve. (P) Interior view of left valve. (R) Hinge plate and resilifer of left valve. (S and T) Ventral view of hinge plate and resilifer of left valve. Scale bars: E = 1 mm; A–D, F, I and L–T = 500 µm; and J = 50 µm; H and K = 10 µm.

**Figure 12:**
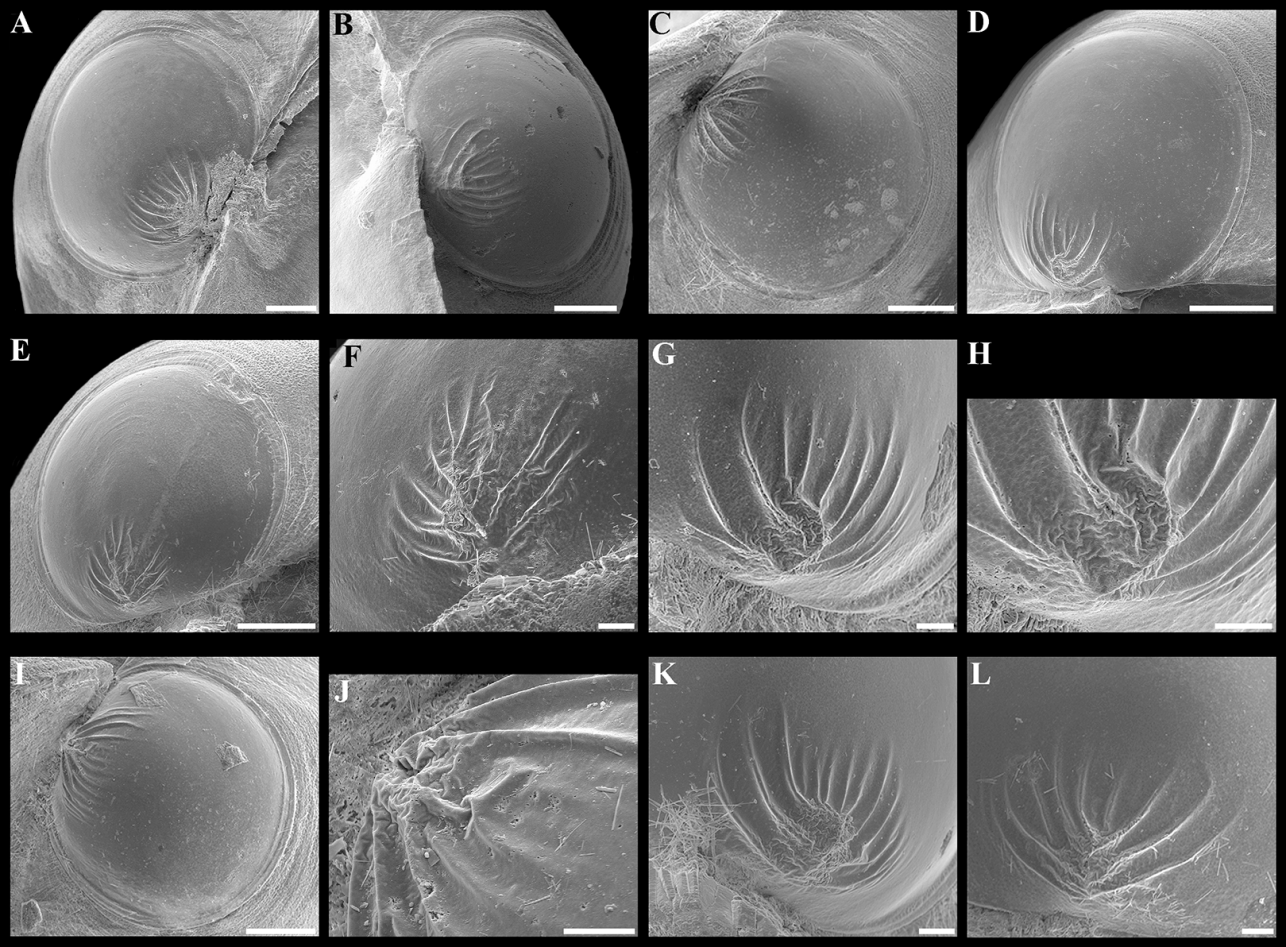
Scanning electron micrographs of prodissoconches of *“Axinulus” oliveri* sp. nov. (A–E and I) Variations in number, shape and position of lamellated folds on surface of prodissoconchs. (F, G, K and L) Lamellated folds. (H and J) Plicate sculpture of ridge from which lamellated folds extend. Scale bars: A–E and I = 50 µm; F–H and J–L = 10 µm.

**Figure 13:**
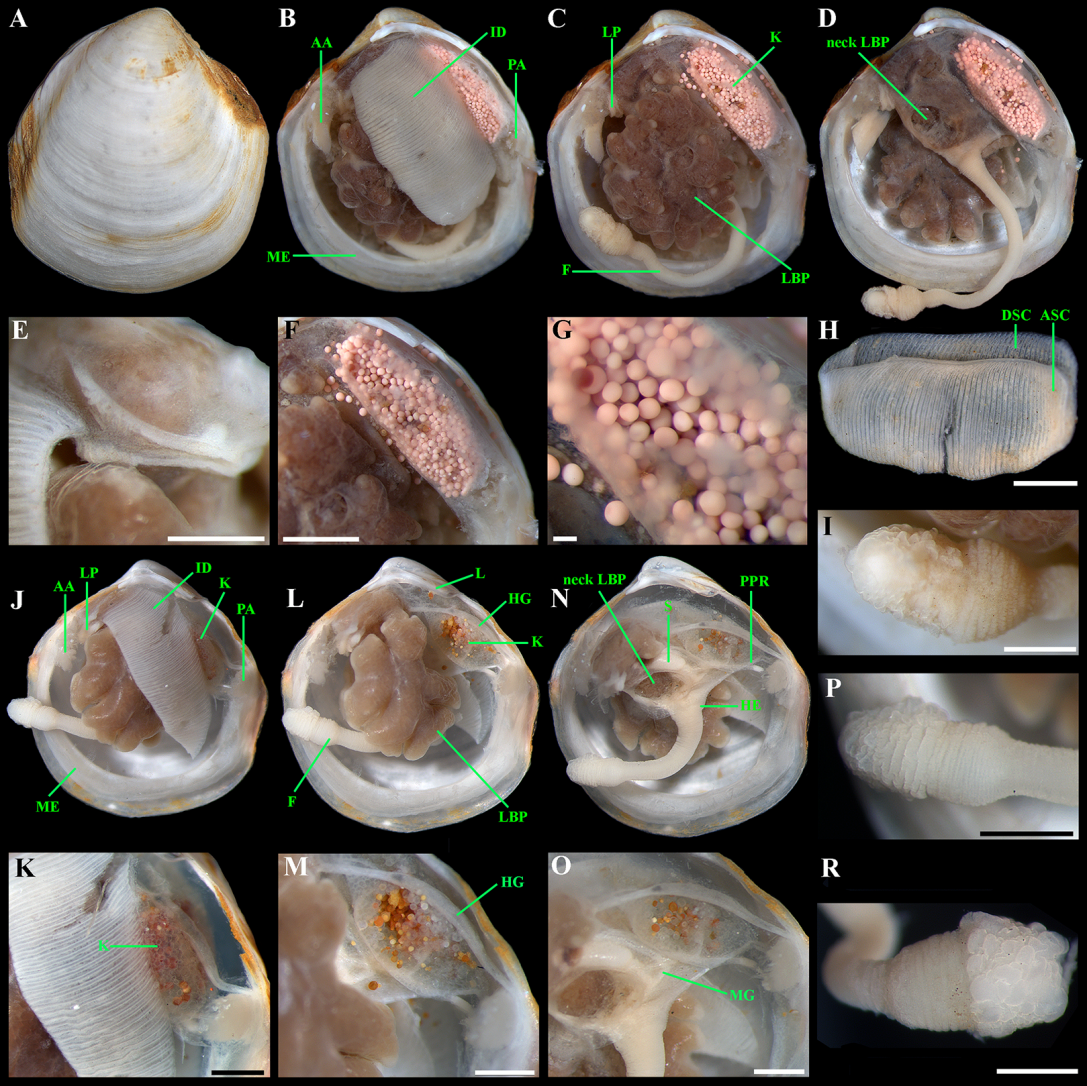
*“Axinulus” oliveri* sp. nov. (A) Exterior view of right valve, shell length 5.4 mm. (B) Gross anatomy after removal of left valve and mantle. (C) Gross anatomy after further removal of left ctenidium. (D) Gross anatomy after further removal of left lateral body pouch. (E) Labial palps. (F) Kidney with pink granules. (G) Kidney granules. (H) Ctenidium. (I) Bulbous of foot. (J) Gross anatomy after removal of left valve and mantle, shell length 4.2 mm. (K) Kidney. (L) Gross anatomy after further removal of left ctenidium. (M) Kidney granules and hindgut. (N) Gross anatomy after further removal of left lateral body pouch. (O) Digestive system. (P and R) Bulbous of foot of different specimens. Abbreviations: A, anterior adductor muscle; APR, anterior pedal retractor muscle; ASC, ascending lamella; DSC, descending lamella; F, foot; HE, heal; HG, hind gut; ID, inner demibranch; K, kidney; L, ligament; LBP, lateral body pouch; LP, labial palps; ME, mantle edge; MG, mid gut; PA, posterior adductor muscle; PPR, posterior pedal retractor muscle; S, stomach. Scale bars: F and H = 1 mm; E, I, K, M and O–R = 500 µm; G = 100 µm.

**Figure 14:**
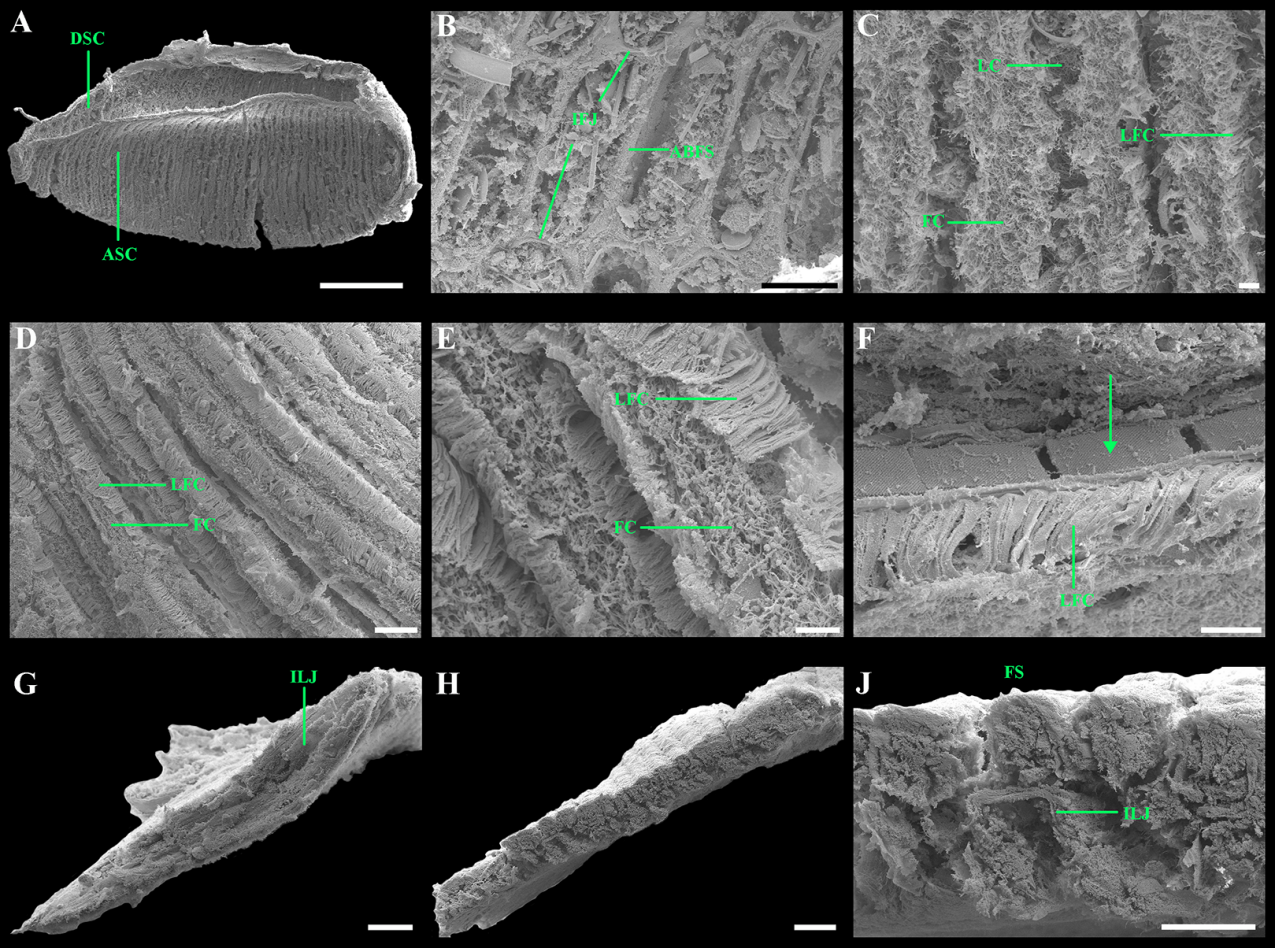
Scanning electron micrographs of the ctenidium of *“Axinulus” oliveri* sp. nov. (A) Inner demibranch. (B) Abfrontal surface of filaments showing inter-filamentar junctions. (C) Ciliation of frontal surface of ascending lamellae. (D) Frontal surface and filaments of descending lamellae. (E) Ciliaton of frontal surface of descending lamellae. (F) Frontal surface with cilia removed (arrow indicates scars on epithelial surfaces). (G) Transverse section of ventral part of inner demibranch showing inter-lamellar junction. (H and J) Cross section of inner demibranch showing inter-lamellar junctions. Abbreviations: ABFS, abfrontal surface; ASC, ascending lamella; DSC, descending lamella; FC, frontal cilia; FS, frontal surface; IFJ, inter-filamentar junction; ILJ, inter-lamellar junction; LC, lateral cilia; LFC, lateral frontal cirri. Scale bars: A = 500 µm; G and H = 100 µm; B, D and J = 50 µm; C, E and F = 10 µm.

***“Axinulus” oliveri* sp. nov.**

(Figs. 10–14, Tables 4 and 5)

**Table 4:** Additional material of “*Axinulus” oliveri* sp. nov. examined in the present study.

Ship, cruise no.	Station	Date	Start		End		Depth (m)	Gear	*N*
			Latitude °N	Longitude °E	Latitude °N	Longitude °E			
Abyssal plain adjacent to the Kuril–Kamchatka Trench, Pacific Ocean									
*Vityaz* 19	3102	20.08.1954	41°16′	147°27.7′	–	–	5,210	OG	2
	3145	18.09.1954	50°59.8′	159°53.3′	–	–	4,927	OG	1
*Sonne* 223	9-9	23–24.08.2012	40°35.49′	150°59.92′	40°34.25′	150°59.91′	5,399–5,398	EBS	1
	9-10	24.08.2012	40°36.13′	151°00,07′	40°35.31′	151°00,12′	5,406–5,404	AGT	2
	9-11	24.08.2012	40°35.12′	151°00,15′	40°34,44′	151°00,15′	5,401–5,404	AGT	1
	10-4	25.08.2012	41°12.02′	150°05.76′	–	–	5,429	GKG	1
	10-9	26.08.2012	41°12.47′	150°05.64′	41°11.18′	150°05.61′	5,252–5,249	EBS	1
	10-12	27–28.08.2012	41°11.70′	150°05.56′	41°13.03′	150°05.71′	5,250–5,255	EBS	1
	11-4	29.08.2012	40°12.86′	148°05.92′	–	–	5,348	GKG	1
	11-5	29.08.2012	40°12.86′	148°06.02′	–	–	5,350	GKG	1
	11-9	30.08.2012	40°13.26′	148°06.24′	40°12.37′	148°05.43′	5,348–5,350	EBS	8
	11-10	30.08.2012	40°13.33′	148°06.48′	40°12.53′	148°05.76′	5,348–5,347	AGT	2
	11-12	31.08.2012	40°13.10′	148°06.45′	40°12.10′	148°05.53′	5,351–5,348	EBS	3
	12-4	01.09.2012	39°43.80′	147°10.16′	39°42.49′	147°09.37′	5,224–5,215	EBS	12
	12-5	01.09.2012	39°43.47′	147°10.11′	39°42.54′	147°09.51′	5,229–5,217	AGT	3
Abyssal slope of the Kuril Islands, Pacific Ocean									
*Sonne* 223	3-4	04.08.2012	47°14.32′	154°42.26′	–	–	4,982	GKG	2
	3-5	04.08.2012	47°14.30′	154°42.23′	–	–	4,984	GKG	9
	3-9	05.08.2012	47°13.83′	154°41.88′	47°14.77′	154°43.05′	4,988–4,992	EBS	1
	3-10	05–06.08.2012	47°14.27′	154°42.17′	47°14.94′	154°43.18′	4,977-4986	AGT	1
*Akademik M.A* *Lavrentyev* 71	10-1	27.07.2015	46°06.895′	152°13.498′	46°05.420′	152°13.566′	4,741	GKG	7
	10-5	28.07.2015	46°07.608′	152°10.505′	46°07.310′	152°11.537′	4,648–4,702	EBS	3
	10-7	29.07.2015	46°06.680′	152°13.834′	46°05.897′	152°14.566′	4,739–4,798	EBS	3
	10-8	29.07.2015	46°06.502′	152°14.139′	46°05.775′	152°15.217′	4,729–4,806	AGT	1
*Sonne* 250	87	16.09.2016	45°01.383′	151°05.527′	45°01.651′	151°05.522′	5,496–5,478	EBS	2
Kuril–Kamchatka Trench									
*Sonne* 250	30	27.08.2016	45°56.821′	152°51.185′	45°56.834′	152°50.943′	6,168–6,164	EBS	1

**Note:**

OG, Okean grab (0.25 m^2^); GKG, giant box corer (0.25 m^2^); EBS, epibenthic sledge; AGT, Agassiz trawl; *N*, number of live specimens.

**Table 5:** *“Axinulus” oliveri* sp. nov. Shell measurements (mm), indices and summary statistics of indices.

Depository	*L*	*H*	*A*	LL	EL	*W*	*H*/*L*	*A*/*L*	LL/*L*	EL/*L*	*W*/*L*
Holotype MIMB 40339	5.6	6.2	2.7	3.0	4.2	4.4	1.107	0.482	0.536	0.750	0.786
Paratype MIMB 40341	5.1	5.4	2.1	2.2	3.9	3.5	1.059	0.412	0.431	0.765	0.686
Paratype MIMB 40341	4.3	4.6	1.9	2.0	3.0	3.4	1.070	0.442	0.465	0.698	0.791
MIMB	3.4	3.4	1.7	1.8	2.2	2.2	1.000	0.500	0.529	0.647	0.647
MIMB	5.7	6.2	2.8	2.9	4.1	4.0	1.088	0.491	0.509	0.719	0.702
Paratype MIMB 40340	4.7	5.1	2.0	2.5	3.3	3.3	1.085	0.426	0.532	0.702	0.702
MIMB	4.5	4.8	1.5	2.1	3.1	3.0	1.067	0.333	0.467	0.689	0.667
Paratype MIMB 40340	4.8	4.9	2.1	2.7	3.3	3.2	1.021	0.438	0.563	0.688	0.667
MIMB	4.2	4.6	1.9	2.4	3.0	2.8	1.095	0.452	0.571	0.714	0.667
MIMB	3.5	3.8	1.7	2.0	2.3	1.7	1.086	0.486	0.571	0.657	0.486
Paratype MIMB 40342	4.7	4.7	1.8	2.6	3.6	3.2	1.000	0.383	0.553	0.766	0.681
MIMB	3.2	3.3	1.5	1.8	2.1	2.0	1.031	0.469	0.563	0.656	0.625
MIMB	2.9	3.0	1.3	1.6	2.3	1.7	1.034	0.448	0.552	0.793	0.586
MIMB	4.1	4.3	1.9	2.3	3.1	2.8	1.049	0.463	0.561	0.756	0.683
Paratype MIMB 40342	4.8	5.0	2.0	2.7	3.3	3.5	1.042	0.417	0.563	0.688	0.729
MIMB	4.3	4.8	1.9	2.4	3.1	3.2	1.116	0.442	0.558	0.721	0.744
Paratype MIMB 40342	4.7	5.0	1.8	2.5	3.4	3.2	1.064	0.383	0.532	0.723	0.681
Paratype MIMB 40342	5.0	5.4	2.1	2.5	4.2	3.5	1.080	0.420	0.500	0.840	0.700
MIMB	3.8	3.9	1.8	2.0	2.6	2.4	1.026	0.474	0.526	0.684	0.632
MIMB	3.9	4.0	1.9	2.2	2.6	2.5	1.026	0.487	0.564	0.667	0.641
Paratype MIMB 40342	4.7	5.0	1.8	3.0	3.6	3.3	1.064	0.383	0.638	0.766	0.702
MIMB	4.6	4.7	1.7	2.5	3.2	3.3	1.022	0.370	0.543	0.696	0.717
MIMB	4.2	4.4	1.4	2.0	3.1	2.6	1.048	0.333	0.476	0.738	0.619
Paratype MIMB 40342	4.4	4.8	1.9	2.5	3.1	3.0	1.091	0.432	0.568	0.705	0.682
MIMB	4.1	4.5	1.8	2.3	3.3	2.7	1.098	0.439	0.561	0.805	0.659
MIMB	4.3	4.7	1.7	2.3	3.0	3.0	1.093	0.395	0.535	0.698	0.698
MIMB	5.0	5.5	2.2	2.7	3.7	3.1	1.100	0.440	0.540	0.740	0.620
MIMB	3.5	3.7	1.6	2.0	2.3	2.3	1.057	0.457	0.571	0.657	0.657
MIMB	3.8	3.8	1.6	1.9	2.4	2.5	1.000	0.421	0.500	0.632	0.658
MIMB	2.9	3.2	1.4	1.6	2.1	2.0	1.103	0.483	0.552	0.724	0.690
MIMB	5.4	5.9	2.5	2.5	4.0	4.0	1.093	0.463	0.463	0.741	0.741
Statistics	L	H	A	LL	EL	W	H/L	A/L	LL/L	EL/L	W/L
Mean	–	–	–	–	–	–	1.062	0.434	0.535	0.717	0.676
SE	–	–	–	–	–	–	0.029	0.035	0.032	0.038	0.041
SD	–	–	–	–	–	–	0.034	0.044	0.042	0.049	0.058
Min	–	–	–	–	–	–	1.000	0.333	0.431	0.632	0.486
Max	–	–	–	–	–	–	1.116	0.500	0.638	0.840	0.791

**Note:**

*L*, shell length; *H*, height; *W*, width; *A*, anterior end length; LL, lunule length; EL, escutcheon length.

*Parathyasira* sp. 1: Kamenev, 2015, p. 191.

*Parathyasira* sp. 2: Kamenev, 2018, p. 234.

urn:lsid:zoobank.org:act:81C12294-3300-4DC1-BCF8-9661DC0C4B61

**Type material and locality:** Holotype (MIMB 40339), abyssal plain adjacent to Kuril-Kamchatka Trench, Pacific Ocean (40°13.10′N, 148°06.45′E–40°12.10′N, 148°05.53′E), 5,351–5,348 m, epibenthic sledge, Coll. A. Brandt, 31-VIII-2012 (RV *Sonne*, cruise no. 223, stn. 11–12); paratypes (2) (MIMB 40340) from holotype locality; paratypes (2) (MIMB 40341), abyssal plain adjacent to Kuril-Kamchatka Trench, Pacific Ocean (40°13.26′N, 148°06.24′E–40°12.37′N, 148°05.43′E), 5,348–5,350 m, epibenthic sledge, Coll. A. Brandt, 30-VIII-2012 (RV *Sonne*, cruise no. 223, stn. 11-9); paratypes (6) (MIMB 40342), abyssal plain adjacent to Kuril-Kamchatka Trench, Pacific Ocean (39°43.80′N, 147°10.16′E–39°42.49′N, 147°09.37′E), 5,224–5,215 m, epibenthic sledge, Coll. A. Brandt, 01-IX-2012 (RV *Sonne*, cruise no. 223, stn. 12-4); paratypes (9) (SMF 360674), abyssal plain adjacent to Kuril-Kamchatka Trench, Pacific Ocean (39°43.80′N, 147°10.16′E–39°42.49′N, 147°09.37′E), 5,224–5,215 m, epibenthic sledge, Coll. A. Brandt, 01-IX-2012 (RV *Sonne*, cruise no. 223, stn. 12-4).

**Other material examined:** 71 live specimens (Table 4).

**Diagnosis:** Shell relatively large (to 5.7 mm in length), ovate-rhomboidal, almost equilateral. Sculpture of thin, commarginal ribs forming very weak, commarginal undulations. Shell surface with pitted micro-sculpture. Anterodorsal and posterodorsal shell margins slightly convex. First posterior fold acute. Second posterior fold very weak. Posterior sulcus very weak. Submarginal sulcus long, sharply defining an escutcheon. Escutcheon very long, narrow. Auricle weak, projecting, very long. Lunule as a weak crest, long, broad. Ligament internal, not visible externally. Prodissoconch large (to 220 µm), smooth; initial part with 9–12 thin, slightly curved, lamellated folds, extending from short, plicate ridge. Lateral body pouches very large, extensively lobed.

**Description.** Shell relatively large (to 5.7 mm in length and 6.2 mm in height), ovate-rhomboidal, equivalve, almost equilateral (A/L = 0.434 ± 0.035), white, thin, fragile, translucent, strongly inflated (W/L = 0.676 ± 0.041), slightly angulated, with height almost equal to length (H/L = 1.062 ± 0.029); patches of silty deposit adhering to anterodorsal and posterodorsal shell margins (Fig. 10; Table 5). Periostracum very thin, colorless, translucent, adherent. Sculpture of closely spaced, thin, commarginal ribs forming, very weak, wide, irregular, commarginal undulations; commarginal ribs more closely spaced on shell margins. Shell surface with pitted micro-sculpture of very small (to 5 µm), shallow, densely spaced pits forming small tubercles at shell margins. Beaks small, raised, prosogyrate (Fig. 11). Anterodorsal shell margin slightly convex, sometimes straight, sloping steeply from beaks, forming a weak, broad rounded angle at transition to anterior margin. Anterior shell margin slightly curved, smoothly transitioning to ventral margin. Ventral margin rounded to narrowly rounded, verging on being angulate. Posterodorsal shell margin slightly convex, sloping steeply from beaks, forming a distinct angle at transition to posterior margin. Posterior margin with weak posterior sinus, sometimes straight, forming a rounded angle at transition to ventral margin. First posterior fold acute. Second posterior fold very weak and rounded. Submarginal sulcus long, sharply defining an escutcheon. Escutcheon very long (EL/L = 0.717 ± 0.038), narrow. Auricle weak, projecting, low and very long, almost filling entire escutcheon. Lunule as a weak crest, weakly defined, long (LL/L = 0.535 ± 0.032), broad; boundary ridges very weak. Ligament opisthodetic, internal, not visible externally, thick, relatively short, about one-third the length of escutcheon, lying in shallow, slightly curved, narrow resilifer. Prodissoconch large (length 191–220 µm), distinct, sharply separated from shell, ovate in outline, convex, smooth; initial part with 9–12 thin, narrow, slightly curved, lamellated folds, sometimes bifurcated at base, extending from short, higher and wider, plicate ridge, located in mid-line of prodissoconch (Fig. 12). Hinge plate thin, edentulous, with small swelling and very small flattened peg beneath beak in each valve (Figs. 11M, 11O, 11R and 11T). Interior of valves white, muscle scars indistinct.

*Anatomy:* Mantle thin, margins thickened. Mantle fusion limited to a small inter-connection below the posterior adductor, forming an exhalant aperture. Anterior adductor muscle elongated, curved almost parallel to anterior shell margin. Posterior adductor muscle 2 times shorter than anterior, ovate (Fig. 13). Foot very long, vermiform, with bulbous portion differentiated into two parts (distal part with wart-like surface; proximal part corrugated like foot stem) (Figs. 13I, 13P and 13R); heel small, indistinct; anterior and posterior pedal retractors narrow, long, weakly developed (Fig. 13N).

Labial palps small (to 800 µm in length), triangular, narrow, with distinctly grooved dorsal zone and long oral groove, lying close to anteroventral corner of inner demibranch (Fig. 13E). Alimentary system with short oesoghagus leading to a relatively large, elongate stomach; combined style sac and midgut strongly curved; hind gut forming an anterior, deep, narrow loop producing rounded distinct angle, passing through the heart and running posteriorly dorsal to kidney and posterior adductor muscle, opening at ventral side of posterior adductor muscle (Figs. 13N and 13O). Lateral pouches very large, extensively lobed; numerous terminations short and thick, cloven or single; each pouch connecting to body by a wide neck. Kidneys very large, dorsoventrally elongated along almost entire posterodorsal shell margin; kidney cells large, vacuolated, containing numerous, pink or orange, large (to 130 µm in diameter), different-size granules often well visible through translucent shell (Figs. 13F, 13G, 13K and 13M). Gonad occupying inner side of lateral pouches. Sexes are separate.

Ctenidium very thin, wide, consisting of a single inner demibranch with fully reflected filaments (up to 55 filaments in large specimens) (Figs. 13H and 14A). Demibranchs covering greater part of lateral body pouches. Demibranch consisting of both ascending and descending lamellae; ascending lamellae near four fifths of descending lamellae length; ascending and descending lamellae fused over half their lengths. Adjacent filaments joined by fine inter-filamentar junctions at about every 135–140 µm (Fig. 14B). Filaments narrow, with no expansion of abfrontal tissue. Frontal surfaces of filaments ciliated with long frontal cilia, very long lateral frontal cirri, and short lateral cilia (remaining pattern of scars well visible on epithelial surfaces after removal of ciliated surfaces) (Figs. 14C–14F). Filaments of asceding and descending lamellae joined in ventral part of ctenidium by inter-lamellar junctions (Figs. 14G–14J). Bacteriocytes absent.

**Variability:** Shell shape and proportions vary significantly both with age and in adult individuals (Table 5). In young specimens, in contrast to adults, the shell is less angular, with more curved anterodorsal and posterodorsal margins; the posterior sulcus is almost indiscernible; commarginal undulations are less distinct or absent; lobes of pouches are less developed, formed by deep divisions at the perimeter of pouches. In adult specimens, the proportions of the shell, the length of the lunule and escutcheon, the degree of curving of shell margins, the depth of the posterior sulcus, and the height of the auricle vary (Figs. 10I–10L). Some specimens have a rather angulated and less elongated dorsoventrally shell with less distinct auricle, almost straight posterodorsal margin, straight posterior margin without sinus, and indistinct posterior sulcus. The number, shape, and position of lamellated folds on the surface of the prodissoconch are also variable (Fig. 12).

**Distribution and habitat:** This species was recorded on the abyssal plain adjacent to the Kuril-Kamchatka Trench (39°42,49′N, 147°09,37′E–50°59,8′N, 159°53,3′E), the abyssal slope of the Kuril Islands (Pacific Ocean) (45°01,383′N, 151°05.527′E–47°14,32′N, 154°42,26′E) at 4648-5496 m depth (bottom temperature (6–8 m above bottom) 1.5–1.6 °C, salinity 34.7‰, oxygen 7.71–7.72 ml. l^−1^)), and in the Kuril-Kamchatka Trench (45°56.821′N, 152°51.185′E–45°56.834′N, 152°50.943′E) at 6,168–6,164 m depth (Fig. 9).

**Comparisons:** As was the case of *“A.” roseus* sp. nov., this species strongly differs from all small-sized species of *Axinulus* in having a large and angulated shell with posterior folds, a large prodissoconch with specific sculpture, and extensively lobbed lateral pouches (Table 3). Moreover, from all small species, except *A. alleni*, it differs in long and deep escutcheon and auricle. The shell of *A. alleni* also has long and deep escutcheon and auricle; nevertheless, in contrast to *“A.” oliveri* sp. nov., this species has globular, very swollen unlobed pouches and the tip of foot not well differentiated from the stem. “*Axinulus” oliveri* sp. nov. is fairly close in shell shape and proportions to *A. subequatoria*, which also has an escutcheon and lobed lateral pouches (Payne & Allen, 1991). However, the shell of “*A*.” *oliveri* sp. nov., unlike *A. subequatoria*, is almost twice larger, ovate-rhomboidal in outline, and has an auricle. The new species is most similar in shell shape and proportions to “*A.” roseus* sp. nov. but well differs from it in having more expressed posterior folds and a more distinct posterior sulcus, an auricle, a completely sunken ligament invisible externally, entirely different sculpture of the prodissoconch, less lobed lateral pouches without numerous small lobes, and in lacking a distinct wide and sunken lunule.

**Derivation of name:** The species name honors Dr. P.G. Oliver, a well-known researcher of the Thyasiridae of the World Ocean. His scientific works and expert opinion are of invaluable help in the study of thyasirids of the Pacific Ocean.

**Remarks:** As was the case of “*A.” roseus* sp. nov., the new species is only provisionally placed in the genus *Axinulus*, because it also strongly differs from the type species of the genus *Axinulus*. According to its shell size and morphology and some anatomical features, the new species more corresponds to the genus *Thyasira*. Like *Thyasira flexuosa* (Montagu, 1803), the type of the genus *Thyasira*, “*A.” oliveri* sp. nov. has a distinctly projecting umbo, posterior folds and a posterior sinus, an auricle, a long submarginal sulcus, a distinct escutcheon, as well as large and multilobed lateral pouches (Oliver & Killeen, 2002). However, unlike *Thyasira*, the new species has a ctenidium consisting of a single demibranch. Like “*A.” roseus* sp. nov., “*A”. oliveri* sp. nov. has a shell relatively large for species with a single demibranch and is most similar in shell morphology and ctenidium structure to the type of the genus *Axinulus*. However, I think that “*A.” oliveri* sp. nov. is also extremely unusual due to a combination of morphological and anatomical features; therefore, likewise, I only provisionally place it in the genus *Axinulus*.

The sculpture of the prodissoconch of “*A.” oliveri* sp. nov. differs principally from that of “*A.” roseus* sp. nov. and much resembles the sculpture of the prodissoconch of *Thyasira patagonica* Zelaya, 2010 and *Parathyasira magelanica* (Dall, 1901) (Zelaya, 2009, 2010). A similar sculpture of prodissoconch was observed in the species provisionally identified as *Parathyasira* sp. that was found on the bottom of the deep-sea basin of the Sea of Japan (Kamenev, 2013). As a rule, the disposition and number of lamellated folds markedly differ among species, which can be considered a clear distinguishing character. However, the sculpture of the prodissoconch is sometimes very similar in different species, for example, in *P. magelanica* and *“A.” oliveri* sp. nov., although these species strongly differ in shell morphology, anatomy, and habitat conditions. It should be remarked that the number and shape of lamellated folds and their disposition on the prodissoconch of “*A.” oliveri* sp. nov. varied markedly. In such a case, when comparing with other close species, prodissoconch sculpture can be used as an additional distinguishing character only if it principally differs from the sculpture of the prodissoconch of the compared species, as in “*A.” roseus* sp. nov.

*“Axinulus” oliveri* sp. nov. was found in many samples collected in a vast region of the northwestern Pacific from northern Japan to the southeastern coast of Kamchatka. This species was mainly represented by a small number of specimens in the samples and probably forms sparse populations on the oceanic plain and slopes of the Kuril Islands. However, its population density sometimes reached 36 ind. m^−2^. Most likely, it is a predominantly abyssal species, which is widespread in the abyssal zone of the northern Pacific Ocean and penetrates into the upper hadal zone (depths of more than 6,000 m) of the Kuril-Kamchatka Trench. It is known that the depth range of 6,000–7,000 m is a zone of transition between abyssal and hadal faunas (Belyaev, 1989; Kamenev, 2019). These depths are the lower boundary of the vertical distribution of many abyssal species of bivalves in the Kuril-Kamchatka Trench (Kamenev, 2019). It is not ruled out that “*A.” oliveri* sp. nov. will be found on the abyssal plain adjacent to the Japan and Aleutian trenches and in the upper part of these trenches, as well as, possibly, off the western coast of the American continent.

**Genus** *Parathyasira* Iredale, 1930

Type species: *Parathyasira resupina* Iredale, 1930

**Diagnosis:** Shell small to medium (<20 mm), fragile, ovate-rhomboidal to rhomboidal, slightly higher than length, subequilateral to equilateral. First posterior fold absent. Second posterior fold weak. Posterior sulcus indistinct. Submarginal sulcus well-defined. Auricle absent. Hinge edentulous. Ligament opisthodetic, internal, weak, in a shallow resilifer. Ctenidium with two demibranchs, lateral body pouches multilobed, foot vermiform.

**Remarks:** Payne & Allen (1991) noted that *Parathyasira*, unlike *Thyasira*, appears to consist of a relatively diverse assemblage of shell morphologies. However, in contrast to *Thyasira*, the shell of *Parathyasira* is higher than long, subquadrate to rhomboidal in outline, with flat or weakly sulcated posterior area and with prominent beaks situated on approximately the mid-line. Moreover, in *Parathyasira*, the first posterior fold and auricle are lacking, the second posterior fold is weak and rounded, the posterior margin is angled and truncated but not distinctly sinuate, submarginal sulcus is long, with almost vertical margins and outlines a deeply incised escutcheon (Payne & Allen, 1991; Oliver & Killeen, 2002; Zelaya, 2009). However, the type species of *Parathyasira* has a microsculpture of radial rows of short spines. Such microsculpture of shell is lacking in many species assigned to *Parathyasira*. Oliver (2015) draw attention to this morphological feature of the type of *Parathyasira* and limited the diagnosis of the genus *Parathyasira* only to species having a similar microsculptute of the shell. Nevertheless, to date the genus *Parathyasira* is comprised of both smooth and radially sculptured species, which, as Oliver & Rodrigues (2017) think, reflects the current unsatisfactory generic definitions within the Thyasiridae. Likewise, I think that generic placement of various species of the genus *Parathyasira* will be clarified following further research.

The genus *Parathyasira* currently includes 10 species (WoRMS Editorial Board, 2020). Huber (2015) transferred three species of *Parathyasira* (*Parathyasira granulosa* (Monterosato, 1874), *P. kaireiae*, and *P. subcircularis*) to the genus *Thyasira*. *Parathyasira granulosa* and *P. subcircularis* are retained in *Thyasira* by Huber (2015), in disagreement with Oliver (2015). Following the viewpoint of Oliver (2015) I think that these species should be retained within the genus *Parathyasira*. Numerous publications provide fairly thorough descriptions of all species of *Parathyasira* and photos of type specimens, which make it possible to perform a correct comparative analysis when describing a new species (Smith, Thomson & Murray, 1885; Verrill & Bush, 1898; Dall, 1901; Iredale, 1930; Nicol, 1965; Lubinsky, 1976; Payne & Allen, 1991; Oliver & Killeen, 2002; Zelaya, 2009; Oliver, 2015).

**Figure 15:**
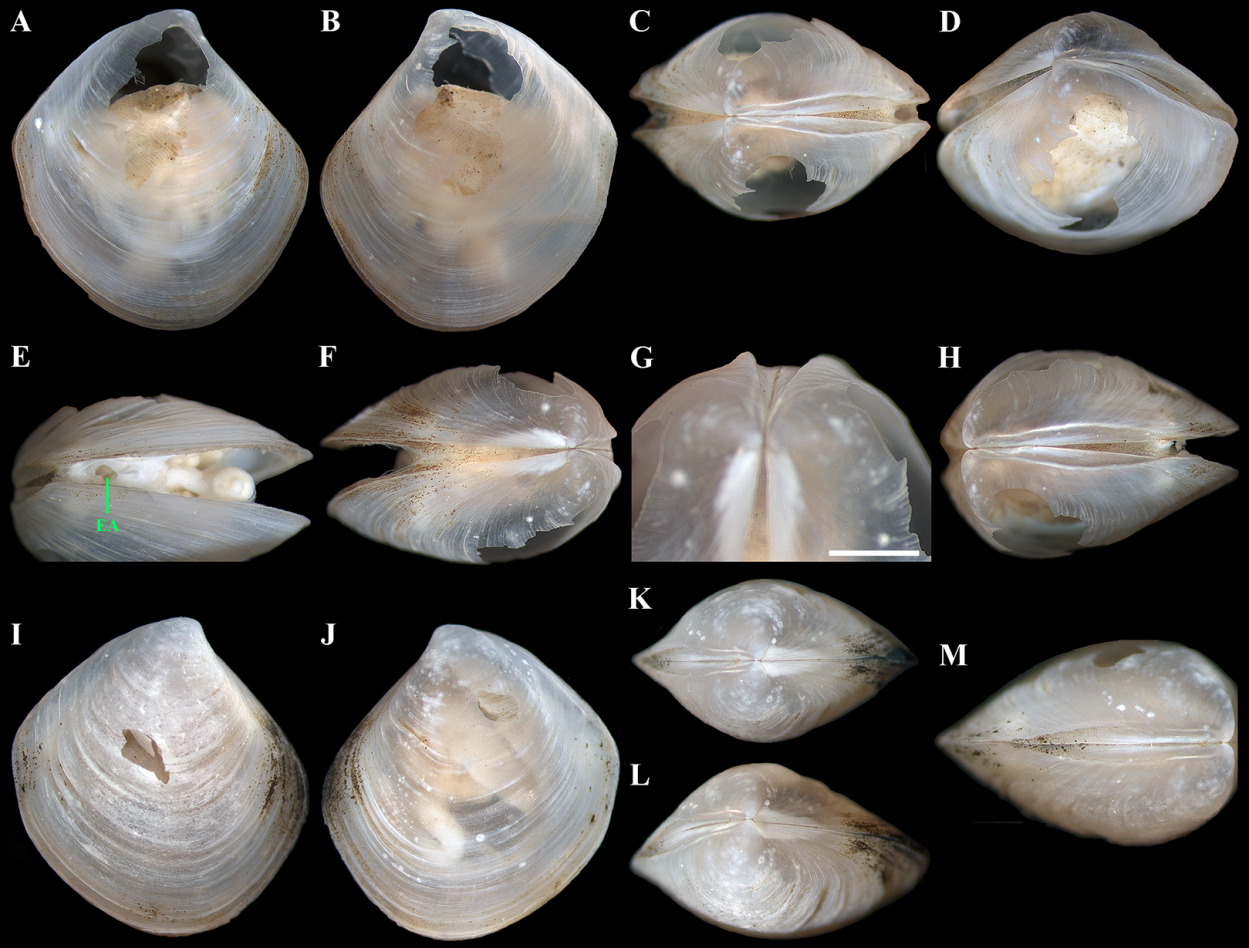
*Parathyasira fragilis* sp. nov. (A–H) Holotype (MIMB 40342): (A–D), exterior, dorsal, and oblique dorsal views of both valves showing lunule and escutcheon, shell length 6.2 mm; (E) posterior view of both vaves showing exhalant aperture; (F and G) anterodorsal views of both valves showing shape of lunule, submarginal sulcus and surface of escutcheon; (H) posterodorsal view of both valve showing shape of escutcheon. (I–M) Specimen from stn. 9-9, exterior, dorsal, oblique dorsal, and posterodorsal views of both valves showing lunule and escutcheon, shell length 4.8 mm. Abbreviation: EA, exhalant aperture. Scale bar: G = 1 mm.

**Figure 16:**
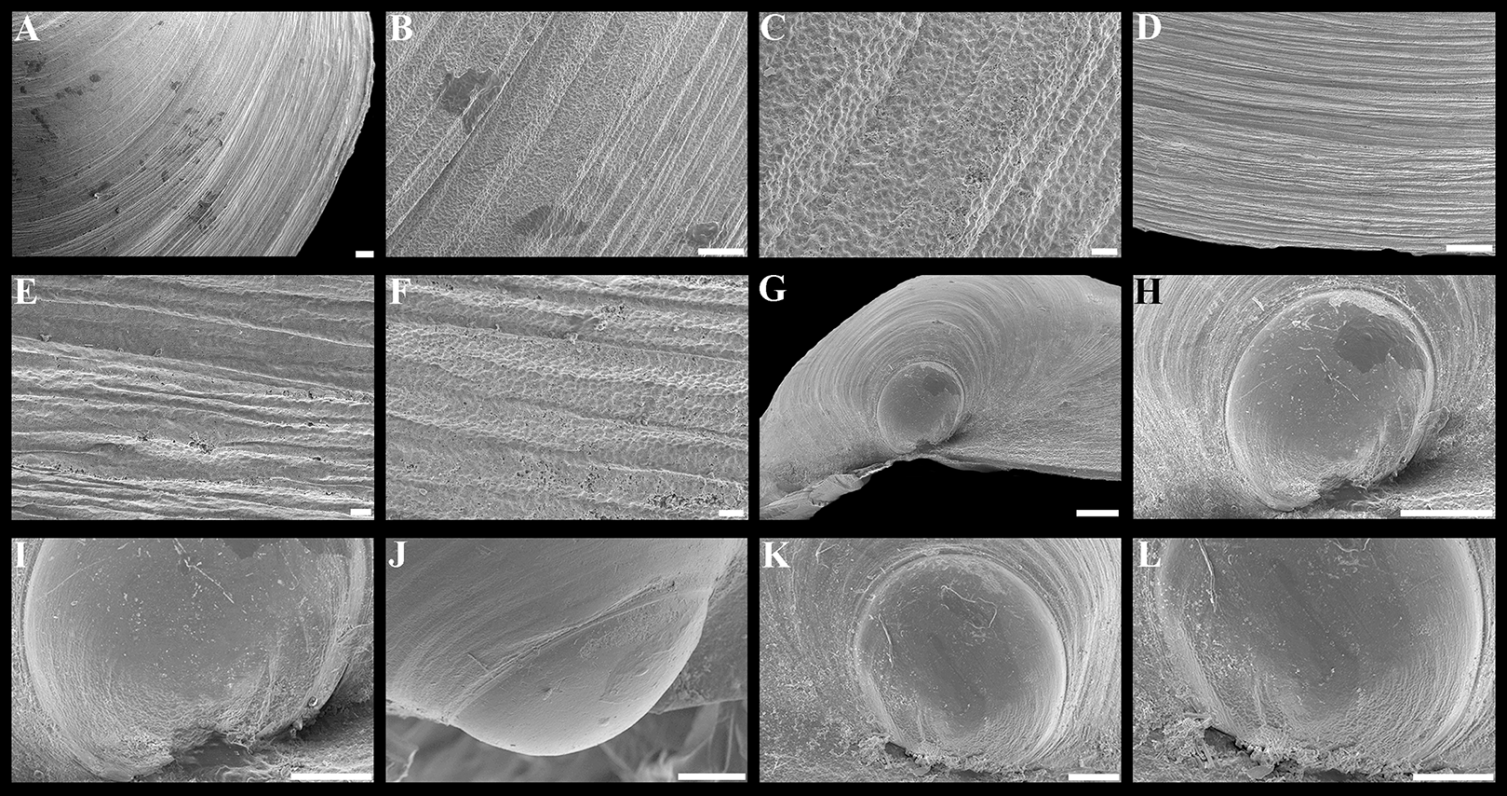
Scanning electron micrographs of *Parathyasira fragilis* sp. nov. (A–C) Sculpture of posterior region of shell. (D–F) Sculpture of ventral shell part. (G–J) Prodissoconch of left valve. (K and L) Prodissoconch of right valve. Scale bars: A, D, G and H = 100 µm; B and I–L = 50 µm; C, E and F = 10 µm.

**Figure 17:**
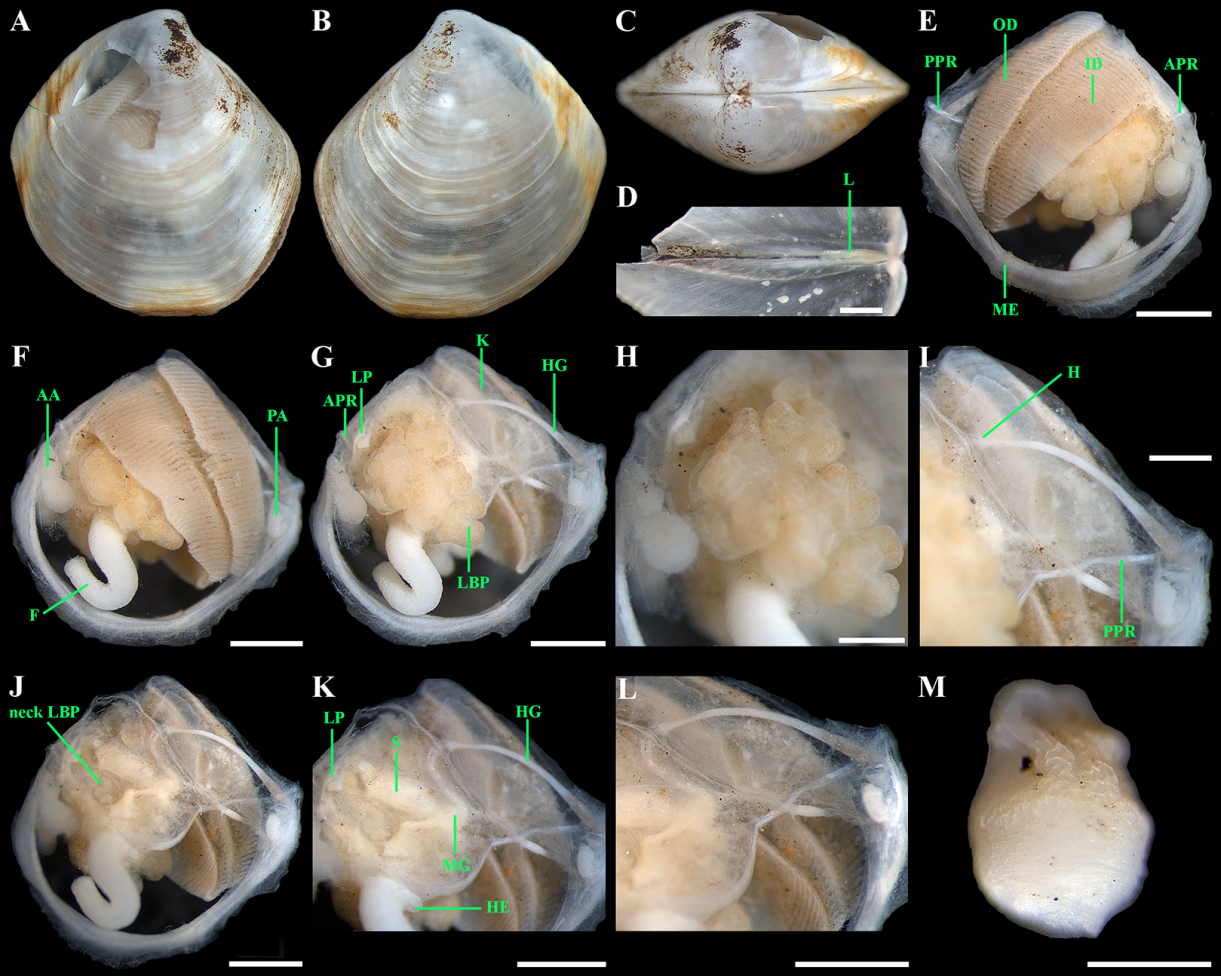
*Parathyasira fragilis* sp. nov. (A–D) Specimen from stn. 10-9, shell length 4.2 mm: (A–C) exterior and dorsal views of both valves showing lunule and escutcheon; (D) ventral view of ligament. (E) Gross anatomy specimen from stn. 9-9 after removal of right valve and mantle (foot without bulbous distal section), shell length 4.8 mm. (F) Gross anatomy after further removal of left valve and mantle. (G) Gros anatomy after further removal of left ctenidium. (H) Left lateral body pouch. (I) Kidney. (J) Gross anatomy after further removal of left lateral body pouch. (K and L) Digestive system. (M) Bulbous distal section of foot. Abbreviations: A, anterior adductor muscle; APR, anterior pedal retractor muscle; ASC, ascending lamella; DSC, descending lamella; EA, exhalant aperture; F, foot; H, heart; HE, heal; HG, hind gut; ID, inner demibranch; K, kidney; L, ligament; LBP, lateral body pouch; LP, labial palps; ME, mantle edge; MG, mid gut; PA, posterior adductor muscle; PPR, posterior pedal retractor muscle; S, stomach. Scale bars: E–G and J–L = 1 mm; D, H, I and M = 500 µm.

***Parathyasira fragilis* sp. nov.**

(Figs. 15–17; Table 6)

**Table 6:** *Parathyasira fragilis* sp. nov. Shell measurements (mm) and indices.

Depository	*L*	*H*	*A*	LL	EL	*W*	*H*/*L*	*A*/*L*	LL/*L*	EL/*L*	*W*/*L*
Holotype MIMB 40343	6.2	6.5	2.6	3.5	4.3	3.6	1.048	0.419	0.565	0.694	0.581
MIMB	4.2	4.5	1.7	1.8	2.5	2.3	1.071	0.405	0.429	0.595	0.548
MIMB	4.8	5.2	1.8	2.8	3.3	2.8	1.083	0.375	0.583	0.688	0.583

**Note:**

*L*, shell length; *H*, height; *W*, width; *A*, anterior end length; LL, lunule length; EL, escutcheon length.

urn:lsid:zoobank.org:act:5B788543-04D5-497A-991B-3C15CD16382E

**Type material and locality:** Holotype (MIMB 40343), abyssal plain adjacent to Kuril-Kamchatka Trench, Pacific Ocean (41°12.47′ N, 150°05.64′ E–41°11.18′ N, 150°05.61′ E), 5,252-5,249 m, epibenthic sledge, Coll. A. Brandt, 26-VIII-2012 (RV *Sonne*, cruise no. 223, stn. 10-9).

**Other material examined:** One live specimen from holotype locality; one live specimen, abyssal plain adjacent to Kuril-Kamchatka Trench, Pacific Ocean (40°35.49′ N, 150°59.92′ E–40°34.25′ N, 150°59.91′ E), 5399-5398 m, epibenthic sledge, Coll. A. Brandt, 23-24-VIII-2012 (RV *Sonne*, cruise no. 223, stn. 9-9).

**Diagnosis:** Shell relatively large (to 6.2 mm in length), rhomboidal, subequilateral, very fragile. Sculpture of closely spaced, commarginal ribs forming weak, commarginal undulations. Shell surface with pitted micro-sculpture. Anterodorsal shell margin straight, very long, descending below mid-point of shell. Posterodorsal shell margin long, slightly convex. Escutcheon very long, narrow, lanceolate. Lunule very long, wide, flat. Ligament internal, sometimes only slightly visible externally, short. Prodissoconch large (length 232 µm), slightly expanded and slightly drawn out anteriorly, irregularly convex, flattened at anterior end; sculpture of two radial, rounded ridges in anterodorsal part and numerous, very fine, irregular folds in posterodorsal part.

**Description.** Shell relatively large (to 6.2 mm in length and 6.5 mm in height), rhomboidal, slightly drawn out anteriorly, equivalve, subequilateral, grayish-white, thin, very fragile, translucent, inflated (W/L = 0.548–0.583), slightly higher than length (H/L = 1.048–1.083); patches of silty and ferruginous deposit adhering to shell margins (Fig. 15; Table 6). Periostracum very thin, grayish, translucent, adherent. Sculpture of closely spaced, conspicuous, commarginal, narrow ribs forming weak, narrow, thin, irregular, commarginal undulations; commarginal ribs more distinct and more closely spaced on shell margins (Fig. 16). Shell surface with pitted micro-sculpture of very small (to 5 µm), shallow, densely spaced pits forming small tubercles at shell margins (Figs. 16C and 16F). Beaks small, raised, prosogyrate, slightly anterior to midline (A/L = 0.375–0.419). Anterodorsal shell margin straight, very long, sloping very steeply from beaks, descending below mid-point of shell. Anterior shell margin strongly rounded. Ventral margin strongly curved, slightly angulate at ventral extremity. Posterodorsal shell margin long, slightly convex, sloping very steeply from beaks, forming a distinct angle at transition to posterior margin. Posterior margin straight or with very weak posterior sinus, forming a very weak, rounded angle at transition to ventral margin. First posterior fold absent. Second posterior fold weak and strongly rounded. Posterior sulcus faint and shallow, indistinct. Submarginal sulcus long, deeply incised with almost vertical margins, not forming a marginal sinus, sharply defining an escutcheon. Escutcheon very long (EL/L = 0.595–0.694), narrow, deep, lanceolate, slightly widening posteriorly, flattened. Auricle absent. Lunule very long (LL/L = 0.429–0.583), wide, flat, weakly defined; boundary ridges very weak, extending along anterodorsal shell margin from beaks to anterior shell margin. Ligament opisthodetic, internal, sometimes only slightly visible externally, thin, short, about one-fourth the length of the escutcheon. Prodissoconch large (length 232 µm), distinct, sharply separated from shell, ovate, slightly expanded and slightly drawn out anteriorly, irregularly convex, flattened at anterior end; sculpture of two radial, wide, rounded ridges of varying length (anteriorly placed ridge almost 3 times longer than posteriorly placed) in anterodorsal part of prodissoconch and numerous, very fine, irregular folds in posterodorsal part of prodissoconch (Figs. 16G–16L). Surface of prodissoconch with closely spaced, shallow pits in folded area; rest of prodissoconch smooth. Hinge plate very thin, edentulous. Muscle scars indistinct.

*Anatomy:* Mantle thin, margins thickened. Mantle fusion limited to a small inter-connection below the posterior adductor, forming an exhalant aperture (Fig. 15E). Anterior adductor muscle elongated, expanding ventrally, divided into three blocks. Posterior adductor muscle small, 3 times shorter than anterior, ovate. Foot long, vermiform, with a muscular ring at the junction with the visceral mass; bulbous portion of foot differentiated into two parts (distal part with wart-like surface; proximal part corrugated like foot stem); heel small, distinct; anterior and posterior pedal retractors narrow, long, weakly developed (Fig. 17).

Labial palps small (to 0.5 mm in length), narrow, triangular. Alimentary system with short oesoghagus leading to a relatively large, elongate stomach; combined style sac and midgut strongly curved; hind gut forming an anterior loop dorsal to style sac, passing through the heart and running posteriorly dorsal to kidney and posterior adductor muscle, opening at ventral side of posterior adductor muscle (Figs. 17J–17L). Lateral pouches relatively small, extensively lobed; lobes short, globular; each pouch connecting to body by a narrow neck (Figs. 17H and 17J). Kidneys large, dorsoventrally elongated, occupying a posterodorsal position between posterior adductor muscle and heart, transparent, almost without granules (Figs. 17G and 17I).

Ctenidium thin, consisting of both inner and outer demibranchs with fully reflected filaments (up to 45 filaments in specimen with shell length of 4.8 mm); outer demibranch about half the height of the inner; descending lamellae of inner demibranch slightly shorter than ascending lamellae; descending lamellae of outer demibranch reduced. Ventral margin of inner demibranch covering dorsal lobes of lateral body pouches (Figs. 17E and 17F).

**Variability:** I examined only 3 specimens of this species with different shell size. Shell shape and proportions varied little among these specimens. Compared to the holotype, the anterodorsal margin in smaller specimens (Figs. 17A–17C) is slightly curved, the ventral margin is slightly angulate, and the posterior sulcus is almost invisible.

**Distribution and habitat:** This species was recorded on the abyssal plain adjacent to the Kuril-Kamchatka Trench (40°34,25′N, 150°59,91′E–41°12,47′N, 150°05,64′E) at 5,249–5,399 m depth (bottom temperature (6–8 m above bottom) 1.5–1.6 °C, salinity 34.7%, oxygen 7.71–7.72 ml. l^−1^) (Fig. 9).

**Derivation of name:** The specific name “fragilis” (adjective in the nominative singular) emphasizes a characteristic feature of the new species, its extremely fragile and thin shell.

**Comparison:** The new species well differs from all species of *Parathyasira* in having a dorsoventrally elongate, very thin, very fragile, rhomboidal shell with long anterodorsal margin, a long, wide, flat, non-excavated lunule, and a large prodissoconch with unique sculpture (Table 7). Moreover, such species as *P. resupina*, *Parathyasira neozelanica* Iredale, 1930, *Parathyasira verconis* (Cotton & Godfrey, 1938), *P. granulosa*, *P. subcircularis*, and *Parathyasira bamberi* P. G. Oliver, 2015 have a shell microsculpture consisting of calcareous spines arranged in dense radial rows (Oliver, 2015), but the new species lacks this microsculpture. A shallow-water arctic *Parathyasira dunbari* (Lubinsky, 1976) also has a dorsoventrally elongated shell with very long anterodorsal margin. However, in contrast to *P. fragilis* sp. nov., this species has a pyriform, strongly asymmetric, solid shell with concave anterodorsal margin, a deeply excavated, cordate, well defined lunule, and a small prodissoconch (160 µm) (Lubinsky, 1976).

**Table 7:** Main differentiating characters of *Parathyasira* species lacking a shell microsculpture of calcareous spines.

Species	Maximum shell length and height (mm)	Shell	Sculpture	Anterodorsal margin	Posterodorsal margin	Lunule	Ligament	Prodissoconch length (µm) and sculpture	References
*Parathyasira fragilis*	*L* = 6.2; *H* = 6.5	Rhomboidal; very thin and fragile, translucent	Conspicuous, commarginal, narrow ribs and weak, irregular undulations; microsculpture of densely spaced pits	Very long, straight, sloping very steeply from beaks, descending below mid-point of shell	Long, slightly convex, sloping very steeply from beaks	Weakly defined, very long, wide, flat	Slightly visible externally, short	232; initial part sculptured with 2 radial, wide, rounded ridges and closely spaced shallow pits.	Present study
*Parathyasira magellanica*	*H* = 7.5	Subquadrate; translucent	Fine, low growth lines	Short, slightly convex or straight, sloping gradually	Long, slightly convex, sloping steeply from beaks	Weakly defined, small, narrow	Visible externally, long	120; initial part sculptured with 5–7 lamellated folds, sometimes bifurcated at base, symmetrically distributed with respect to main central axis	Dall (1901) and Zelaya (2009)
*Parathyasira dearborni*	*H* = 6.0	Rhomboidal; thin	Commarginal ribs composed by microscopic irregular corrugations and pustules	Long, straight or slightly concave, sloping steeply	Long, slightly convex, sloping steeply from beaks	Weakly defined, short	Visible externally, long	115; initial part sculptured with 14 strong folds, bifurcated at base, symmetrically distributed with respect to main central axis	Nicol (1965) and Zelaya (2009)
*Parathyasira dunbari*	*L* = 6.5 *H* = 8.6	Pyriform; high, strongly asymmetric, solid	Conspicuous, commarginal ribs	Very long, concave, sloping very steeply from beaks, descending below mid-point of shell	Long, convex, sloping very steeply from beaks	Well defined, large, strongly excavated, cordate	Visible externally, long	160; smooth	Lubinsky (1976) and Oliver & Killeen (2002)
*Parathyasira equalis*	*H* = 8.0	Ovate to diamond shaped; solid	Weak commarginal lines and growth stops, frequently with irregular dents, weak ridges and fine radial striae	Short, concave or straight, sloping steeply from beaks	Long, convex, sloping steeply from beaks	Weakly defined, small, weakly excavated	Not visible externally, long	155–167; series folds or wrinkles radiating from the anterior end to apex	Verrill & Bush (1898), Payne & Allen (1991) and Oliver & Killeen (2002)
*Parathyasira biscayensis*	*L* = 8.2 *H* = 9.1	Rhomboidal; translucent	Irregular indented commarginal growth lines	Long, almost straight, sloping steeply from beaks	Long, convex, sloping steeply from beaks	No data	Long	No data	Payne & Allen (1991)
*Parathyasira marionensis*	*L* = 4.0 *H* = 4.5	Subquadrate	Fine, commarginal ribs	Long, concave, sloping gradually from beaks	Long, convex, sloping steeply from beaks	Short, narrow	No data	No data	Smith, Thomson & Murray (1885)

**Note:**

*L*, shell length; *H*, shell height.

**Remarks:** The shell of the new species lacks a microsculpture of radial rows of short spines. However, I place this species in the genus *Parathyasira*, because it is most similar in shell morphology to the type of the genus *Parathyasira*, and all of the rest of its main morphological features agree with the diagnosis of the genus.

*Parathyasira fragilis* sp. nov. is currently the deepest-water species of the genus *Parathyasira*. Most species of the genus were recorded in the shelf and bathyal zones. Only *P. bamberi* and *Parathyasira equalis* (Verrill & Bush, 1898) were found in the abyssal zone at depths of 3,356 m and down to 4,734 m, respectively (Payne & Allen, 1991; Oliver, 2015). In addition, a large species of thyasirid was found in the Japan Trench at 5,343–6,390 m depth and described as *P. kaireiae* (Okutani, Fujikura & Kojima, 1999; Fujikura et al., 2002; Okutani & Fujikura, 2002). Subsequently, the species was transferred to the genus *Thyasira* (Okutani, 2000). However, I think that the shell morphology of *T. kaireiae* is more consistent with the genus *Parathyasira* and that this interesting species needs further investigation.

## Discussion

Payne & Allen (1991) think that one of the adaptations of thyasirids for life at great depths of the World Ocean is a significant decrease in the shell size, compared to shallow-water species, as well as the presence of one instead of two ctenidial demibranchs. All species without outer demibranch invariably had a shell length of less than 4 mm. Likewise, all thyasirid species found in the Atlantic Ocean at abyssal depths have small shells (less than 5 mm in length) (Payne & Allen, 1991; Allen, 2008). However, *P. bamberi* that was found in the Arabian Sea at a depth of 3,356 m has a very large shell with a length of 18.7 mm, compared to Atlantic deep-sea species (Oliver, 2015). A large species *Channelaxinus excavata* (Dall, 1901) with a shell length of up to 24 mm (Coan, Scott & Bernard, 2000) inhabits the bottom of the deep-sea basin of the Sea of Okhotsk at depths of more than 3,000 m (Kamenev, 2018). *Axinus cascadiensis* Oliver & Holmes, 2007 with a shell length up to 32 mm was collected from a hydrothermal spring site at Baby Bare Seamount, Cascadia Basin (North-East, NE Pacific Ocean) at a depth of 2,592 m (Oliver & Holmes, 2007). In addition, the species *A. hadalis* and *T. kaireiae* found at hadal depths (more than 6,000 m) of the Japan Trench have a large shell of 36.5 mm and 12.3 mm in length, respectively (Okutani, Fujikura & Kojima, 1999). All these species, except *T. kaireiae*, have a large ctenidium consisting of two demibranchs with fully reflected filaments (Okutani, Fujikura & Kojima, 1999; Oliver & Holmes, 2007; Oliver, 2015). It is possible that *T. kaireiae*, whose body anatomy was not studied, also has a large ctenidium with two demibranchs. *Axinulus hadalis, A. cascadiensis*, and *T. kaireiae* were found associated with chemosynthetic communities and the gills of *A. hadalis* and *A. cascadiensis* harbor endosymbiotic bacteria (Fujikura et al., 1999, 2002; Fujiwara et al., 2001; Okutani & Fujikura, 2002; Sasaki, Okutani & Fujikura, 2005; Oliver & Holmes, 2007). The three deep-sea species described in this article also have a relatively large size of the shell and well-developed gills, with either a single demibranch or two demibrnchs. All these species were not associated with chemosynthetic communities; nor endosymbiotic bacteria were found in *“A”. roseus* sp. nov. and *“A”. oliveri* sp. nov., which have a single demibranch. Moreover, I discovered a group of relatively large thyasirids with two gill demibranchs at the abyssal and hadal depths of the northwestern Pacific. Thus, the adaptation of thyasirids for life in the deep sea is not necessarily connected with the decrease in shell size and the reduction of the gills. *“Axinulus” roseus* sp. nov. and *“A”. oliveri* sp. nov. significantly exceed 4 mm in shell length and are currently the largest species with a single demibranch.

Studies of the bivalve material collected in the abyssal and hadal zones of the northwestern Pacific showed that thyasirids are one of the few groups of bivalves that are well adapted to living at great depths. In contrast to the Atlantic (Allen, 2008), the Thyasiridae at depths of more than 5,000 m in the northwestern Pacific is represented by the largest number of species, compared to other bivalve families of bivalves (Kamenev, 2015, 2018, 2019). Payne & Allen (1991) suggested that there are only a few exclusively abyssal thyasirid species that are limited in distribution merely to abyssal depths. Most thyasirids inhabit shallower depths of the bathyal zone and spread to abyssal depths, where they often become one of the dominant (in abundance) taxonomic groups of bivalves. For example, in the Atlantic Ocean, only 10 species of bivalves were dominant in quantitative parameters in epibenthic sledge samples collected at depths of more than 3,000 m. Of these, four species were thyasirids (Allen, 2008).

In the northwestern Pacific, in contrast to the Atlantic, many thyasirid species appear to be exclusively abyssal and hadal species (Okutani, Fujikura & Kojima, 1999; Fujikura et al., 2002; Okutani, 2003; Sasaki, Okutani & Fujikura, 2005; Kamenev, 2015, 2018, 2019). The shelf and bathyal zones of this region have been well explored (Scarlato, 1981; Kamenev, 1995, 2013; Okutani, 2000; Kamenev & Nekrasov, 2012). However, these studies did not reveal thyasirid species which were later found at abyssal depths of the deep-sea basin of the Sea of Okhotsk and on oceanic plains, and on the slopes and bottom of oceanic trenches. Moreover, many thyasirid species form abundant populations at abyssal and hadal depths of the northwestern Pacific and occupy a dominant position in the benthic communities (Belyaev & Mironov, 1977; Belyaev, 1989; Okutani, Fujikura & Kojima, 1999; Fujikura et al., 2002; Kamenev, 2013, 2015, 2018, 2019). To all appearances, “*A.” roseus* sp. nov. is an exclusively hadal species and its populations thrive at the bottom of the Kuril-Kamchatka Trench. It is adapted to live at a depth of more than 9,000 m with a very high hydrostatic pressure, and the feeding and environmental conditions at the bottom of the trench are apparently favorable for this species to occur in large numbers. The low content of organic matter in bottom sediments and bottom water is one of the limiting factors in the distribution of benthic animals in the abyssal and hadal zones of the World Ocean (Rex et al., 2005; Allen, 2008; Smith et al., 2008; Rex & Etter, 2010; Brault et al., 2013; Stuart & Rex, 2017). In addition, the depletion of food supply probably leads to a decrease in body size of many benthic animals (Belyaev, 1989), including deep-sea bivalves, most of which are less than 5 mm in shell length (Allen, 2008). However, the Kuril-Kamchatka Trench is close to the continent and receives large inputs of organic matter via terrigenous runoff. Being located in one of the most productive areas of the World Ocean, it also receives a very large supply of organic matter from the surface ocean (Belyaev, 1989; Itoh et al., 2011; Kitahashi et al., 2012, 2013; Stewart & Jamieson, 2018). Moreover, waters from the Sea of Okhotsk, characterized by a high level of primary production, enter the Pacific Ocean in the region of the Kuril-Kamchatka Trench through numerous straits between the Kuril Islands (Belyaev, 1989; Shuntov, 2001). Therefore, the bottom sediments of the Kuril-Kamchatka Trench have high organic matter content (Belyaev, 1989; Itoh et al., 2011; Jamieson, 2015; Sattarova & Aksentov, 2018; Kamenev, 2019). This creates favorable conditions for the existence of a large number of species of benthic animals in the hadal zone of the trench, many of which are found in very large numbers at its bottom (Belyaev, 1989). *“Axinulus” oliveri* sp. nov. is also not a rare species. It is widespread in a vast region of the northwestern Pacific, where it sometimes forms populations with a relatively high density on the abyssal plain.

The new species of thyasirids described here have a remarkable microsculpture of the prodissoconch, which in many cases is unique and provides a reliable diagnostic feature facilitating identification and separation of species. In *P. magellanica, P. dearborni*, and *T. scotiana*, Zelaya (2009) described a prodissoconch sculpture consisting of prominent radial folds symmetrically arranged along a main central axis. He remarked that such a sculpture of the prodissoconch was for the first time observed in thyasirids, which usually have a smooth prodissoconch surface (Oliver & Killeen, 2002; Oliver & Holmes, 2007). Subsequently, a similar sculpture of prodissoconch was described in *A. antarcticus*, *T. patagonica*, and *Parathyasira* sp. (Zelaya, 2010; Kamenev, 2013). Thus, the prodissoconch of many thyasirid species belonging to different genera has a distinct and often unique sculpture, which undoubtedly should be used for diagnostic and systematic purposes.

The size and morphology of the shell and anatomy of *“A”. roseus* sp. nov. and *“A”. oliveri* sp. nov., except the presence of a single demibranch of the gill, almost completely correspond to the characters of the genera *Parathyasira* and *Thyasira*, respectively. Thus, the main diagnostic characters according to which many species of thyasirids were rather conditionally assigned to *Parathyasira* and *Thyasira*, turned out to be quite universal and thus insufficiently reliable for delimiting species. The universality and great variability of many diagnostic characters creates a big problem for the identification of many species of thyasirids and the systematics of this family. The generic assignment of thyasirid species is frequently problematic. Study and description of a large number of deep-sea thyasirid species found in different regions of the World Ocean (Filatova, 1968; Belyaev & Mironov, 1977; Belyaev, 1989; Allen, 2008; Kamenev, 2013, 2015, 2018, 2019) that have an unusual combination of morphological and anatomical features, as well as new unique features will clarify the systematic position of many species and will help solve a number of systematic problems of this family.

## Additional Information and Declarations

### Data Availability

The following information was supplied regarding data availability: The raw data is available in Figs. 2–8, 10–17 and in Tables 1–7. The type specimens examined in this study are deposited in the Museum of the Institute of Marine Biology, A.V. Zhirmunsky National Scientific Center of Marine Biology, Far Eastern Branch, Russian Academy of Sciences (MIMB), Vladivostok (Russia), under accession numbers MIMB 40337-40343, and the Senckenberg Museum Frankfurt, Frankfurt am Main (Germany) (SMF), under accession numbers SMF 360674, 360675.

### New Species Registration

The following information was supplied regarding the registration of a newly described species: Publication LSID: urn:lsid:zoobank.org:pub:E693F0D2-D228-4E99-A7CD-143A5A7E2B9B. *“Axinulus” roseus* sp. nov. LSID: urn:lsid:zoobank.org:act:28594E9C-C258-4305-A75F-DD2066FDA885. *“Axinulus” oliveri* sp. nov. LSID: urn:lsid:zoobank.org:act:81C12294-3300-4DC1-BCF8-9661DC0C4B61. *Parathyasira fragilis* sp. nov. LSID: urn:lsid:zoobank.org:act:5B788543-04D5-497A-991B-3C15CD16382E.
